# High-quality genome assembly of 'Cuiguan' pear (*Pyrus pyrifolia*) as a reference genome for identifying regulatory genes and epigenetic modifications responsible for bud dormancy

**DOI:** 10.1038/s41438-021-00632-w

**Published:** 2021-09-01

**Authors:** Yuhao Gao, Qinsong Yang, Xinhui Yan, Xinyue Wu, Feng Yang, Jianzhao Li, Jia Wei, Junbei Ni, Mudassar Ahmad, Songling Bai, Yuanwen Teng

**Affiliations:** 1 College of Agriculture and Biotechnology, Zhejiang University, Hangzhou, Zhejiang 310058 China; 2 Key Laboratory for Silviculture and Conservation, Ministry of Education, Beijing Forestry University, Haidian District, Beijing, 100083 China; 3 College of Agriculture, Ludong University, Yantai, Shandong 264025 China; 4 Hainan Institute of Zhejiang University, Sanya, Hainan 572000 China

**Keywords:** Epigenomics, Gene expression profiling, Open reading frames, Transcriptomics

## Abstract

*Dormancy-associated MADS-box* (*DAM*) genes serve as crucial regulators of the endodormancy cycle in rosaceous plants. Although pear *DAM* genes have been identified previously, the lack of a high-quality reference genome and techniques to study gene function have prevented accurate genome-wide analysis and functional verification of such genes. Additionally, the contribution of other genes to the regulation of endodormancy release remains poorly understood. In this study, a high-quality genome assembly for 'Cuiguan' pear (*Pyrus pyrifolia*), which is a leading cultivar with a low chilling requirement cultivated in China, was constructed using PacBio and Hi-C technologies. Using this genome sequence, we revealed that pear *DAM* genes were tandemly clustered on Chr8 and Chr15 and were differentially expressed in the buds between 'Cuiguan' and the high-chilling-requirement cultivar 'Suli' during the dormancy cycle. Using a virus-induced gene silencing system, we determined the repressive effects of *DAM* genes on bud break. Several novel genes potentially involved in the regulation of endodormancy release were identified by RNA sequencing and H3K4me3 chromatin immunoprecipitation sequencing analyses of 'Suli' buds during artificial chilling using the new reference genome. Our findings enrich the knowledge of the regulatory mechanism underlying endodormancy release and chilling requirements and provide a foundation for the practical regulation of dormancy release in fruit trees as an adaptation to climate change.

## Introduction

Bud endodormancy is a complex biological process necessary for the survival and development of perennial plants. During the endodormancy transition, buds undergo substantial internal changes, but there are no visible morphological changes^1,2^. Endodormancy release requires long-term exposure to cold, and the amount of cold needed to resume normal growth in the spring following the winter period is commonly referred to as the chilling requirement (CR). Insufficient chilling accumulation causes physiological disorders with adverse effects on bud sprouting, flowering, and fruit production. Global warming has resulted in insufficient winter chilling accumulation, leading to an unsatisfactory CR of some commercially grown deciduous fruit tree species in specific regions^3,4^. Therefore, elucidating the mechanism controlling the dormancy cycle may help researchers address the problems caused by climate change.

Several lines of evidence have shown that dormancy-associated MADS-box (*DAM*) genes are involved in the regulation of dormancy. DAM proteins belong to the AGAMOUS 24/SHORT VEGETATIVE PHASE (AGL24/SVP) subfamily of MADS-box proteins, and *DAM* genes were first described in *Prunus* spp., of which six tandemly repeated *DAM* genes (*DAM1*–*6*) are located on chromosomes 1^5–7^. The large deletion fragment containing *DAM1*–*4*, which also eliminated the expression of *DAM5* and *DAM6*, was shown to stop the growth cessation of buds of the *evergrowing* (*evg*) peach mutant^5^. Moreover, the ectopic expression of *PmDAM6* in poplar promotes the formation of dormant terminal buds^6^, while *DAM* genes have also been identified in other rosaceous species^8^.

Three *DAM* genes have been characterized in pear, *PpyMADS13-1*, *-2*, and *-3*, also known as *PpyDAM1/2/3*, which were first identified via homologous cloning based on peach *DAM* genes^9,10^. After publication of the 'Suli' genome sequence, Niu et al. analyzed the pear MADS-box family and identified the same *DAM* genes^10^ (*DAM1/2/3* or *MADS13-1/-2/-3*) that enhance endodormancy by inhibiting *FT2* expression^9–11^. Further analysis showed that pear *DAM* genes are regulated by C-repeat binding factors and abscisic acid (ABA)-binding factors (ABFs), which mediate low-temperature-induced dormancy^12,13^. Although the biochemical functions of DAMs have been thoroughly studied in rosaceous fruit tree species, their roles in pear floral buds have not been characterized. In nonrosaceous species, *DAM* genes are usually named *SVP* or *SVP-like* (*SVL*). The poplar SVL protein enhances terminal bud endodormancy by directly promoting the biosynthesis of ABA^14,15^ and activating the expression of the callose synthetase gene *CALLOSE SYNTHETASE 1*. This causes plasmodesmata to close, preventing intercellular communication^15^. Thus, *DAM* genes mainly mediate growth repression during endodormancy.

Like *FLC*s during vernalization, *DAM* genes are thought to be regulated by epigenetic modifications during long-term exposure. Several lines of evidence indicate that H3K4me3 and H3ac are positively associated with *DAM*/*SVP* expression, whereas H3K27me3 is negatively associated with DAM/SVP expression in kiwifruit and *Prunus* spp^16–19^. Recently, a comprehensive analysis of peach *DAM* gene epigenetic regulation showed that multiple epigenetic events, including H3K27me3 and those involving 21-nucleotide small (s)RNAs and noncoding (nc)RNAs, were induced by chilling exposure and that such changes were inversely correlated with *DAM* downregulation^19^. Saito et al.^20^ observed H3K4me3 changes in 'Kosui' pear buds during the transition from endodormancy to ecodormancy, but they did not determine the association between *MADS13-1* expression and H3K27me3. Similarly, *SVL* expression in poplar under chilling exposure was found to be unrelated to H3K27me3 levels^14^. Therefore, the association of epigenetic changes with the expression of *DAM* genes in pear is still unclear.

Pear cultivars with different CRs exhibit different endodormancy characteristics during chilling accumulation. The high-CR cultivar 'Suli' (with a CR of 800–1000 h)^21^ demonstrates a clear deep endodormancy stage (with bud breaking rates of almost 0 under forcing conditions) and obvious endodormancy release following chilling accumulation; conversely, the low-CR cultivar 'Cuiguan' (with a CR of <400 h)^22^ shows no deep endodormancy stage throughout chilling accumulation, and the lowest bud breaking rate under forcing conditions is ~20%. High-CR cultivars prefer low-CR cultivars to understand the biological changes that occur during endodormancy release because of the clear differentiation of endodormancy stages.

Since the first genome assembly of 'Suli' pear (*Pyrus pyrifolia*; white pear group) was published in 2013^23^, the genomes of 'Bartlett' European pear (*P. communis*)^24^, Asian wild pear (*P. betulifolia*)^25^, and a select pear rootstock [(*P. ussuriensis* × *P.* *communis*) × spp.]^26^ have also been released. However, a reference genome for Chinese sand pear, the most widely cultivated *Pyrus* species^27^, is lacking. Additionally, the published genome of 'Suli', which is a representative Chinese white pear cultivar, is of relatively low quality because of technological limitations at the time of its assembly. Therefore, a high-quality genome for cultivated Chinese sand pear is needed.

In this study, a multiomics strategy was adopted to mine the essential genes regulating chilling-induced endodormancy release. We first assembled a high-quality genome of the low-CR pear cultivar 'Cuiguan', which is a popular cultivar in South China. Using this reference genome, we performed a genome-wide analysis of *DAM* genes and compared their expression patterns between pear cultivars with high and low CRs. We also verified DAM functions related to the inhibition of bud break in pear buds using a virus-induced gene silencing (VIGS) system. Because the high-CR cultivar 'Suli' has an obvious endodormancy stage and release process, using the high-quality 'Cuiguan' pear genome, we analyzed the transcriptome and genome-wide H3K4me3 modifications during the artificial chilling accumulation (ACA) period to identify candidate genes potentially involved in regulating endodormancy release. Overall, we obtained a new reference genome for cultivated Asian pear and identified several genes that are potentially involved in the regulation of the dormancy transition during the chilling accumulation period. These data will provide a basis for future investigations into the mechanism regulating endodormancy release in rosaceous fruit trees.

## Results

### De novo assembly of the 'Cuiguan' pear genome sequence

We assembled the *P. pyrifolia* 'Cuiguan' pear genome based on Illumina short reads, PacBio subreads, and Hi-C data using a hierarchical method. Approximately 36.85 Gb of Illumina paired-end short reads (270 bp) were produced, with an estimated heterozygosity ratio of 0.89%. Based on the 17-mer depth distribution of the Illumina reads, we estimated the genome to be 501.34 Mb in length. Next, we generated ~62.45 Gb of PacBio subreads (N50 = 19.07 kb) and used them to construct a genome comprising 694.68 Mb. Then, we used 74.03 Gb of clean Hi-C data to anchor the primary genome sequence. The final assembled genome (541.34 Mb; contig N50 = 1.28 Mb) included 17 pseudochromosomes (Table 1). We aligned the Illumina reads to the assembled genome, resulting in mapping of 98.58% of the reads. These 17 pseudochromosomes represented ~94% of the total assembled sequences and were denoted as the 'Suli' (*P. bretschneideri*) and *P. betulifolia* genomes (Supplementary Fig. S1).

**Table 1:** Information regarding the 'Cuiguan' and 'Suli' pear genomes

	'Cuiguan'	'Suli'
Total assembly size (Mb)	541.3	512.0
Contig number	1103	25312
Contig N50 (kb)	1280.2	35.7
Scaffold number	428	2103
Scaffold N50 (kb)	27968.3	540.8
Repeat sequence (Mb)	314.99	271.9
Gene number	42622	42812

### Annotation and phylogenetic analysis of the 'Cuiguan' pear genome

**Fig. 1:**
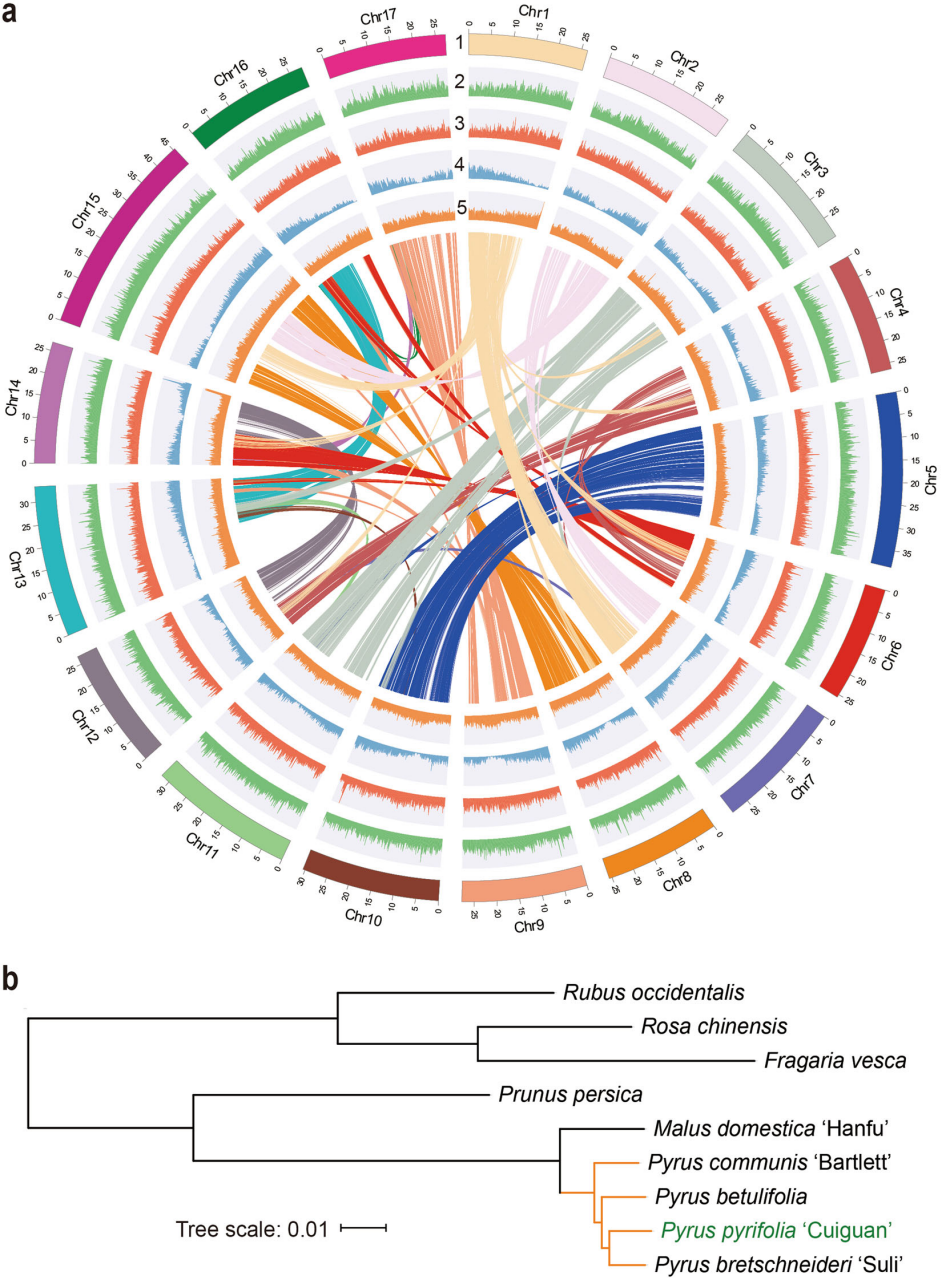
'Cuiguan' pear genome profile and phylogeny. **a** 'Cuiguan' pear genome profile. (1) pseudochromosomes; (2) LTR copia density; (3) LTR gypsy density; (4) gene density; (5) GC content distribution. The colored lines connect gene pairs within syntenic blocks. **b** Phylogenetic tree of 'Cuiguan' pear and eight other rosaceous species. The orange clade includes four *Pyrus* species whose genome has been published

We identified a total of ~314.99 Mb of repetitive elements, occupying ~58.19% of the assembled sequence. We detected LTR copia and LTR gypsy as two of the repetitive elements, which accounted for a large proportion of the total repetitive sequences in the assembled genome (Fig. 1a, Supplementary Table S1). The LTR assembly index (LAI) of the 'Cuiguan' pear genome was 17.62. We combined three gene prediction strategies [ab initio, homology-based search, and RNA sequencing (RNA-seq)] to predict 42,622 protein-coding genes with an average length of 3151 bp. The number of annotated genes was similar to that in the 'Suli' pear genome (Fig. 1a, Table 1). Moreover, 39,929 genes (93.7%) were detected on the pseudochromosomes. We used the BUSCO 3 program^28^ to evaluate the predicted genes based on 1440 highly conserved plant genes. Specifically, 94.8% complete BUSCOs were identified, reflecting the integrity of our genome and the successful prediction of protein-coding genes. These predicted genes were further annotated using the NR, Gene Ontology (GO), EuKaryotic Orthologous Groups (KOG), Kyoto Encyclopedia of Genes and Genomes (KEGG), and TrEMBL databases, and 99.14% (42,257) of the genes were successfully annotated (Supplementary Table S2).

We used 510 single-copy gene families from the following species to construct a phylogenetic tree according to the maximum likelihood method (Fig. 1b): 'Cuiguan' pear (*P. pyrifolia*), 'Suli' pear (*P. bretschneideri*)^23^, *P. betulifolia*^25^, *P. communis*^24^, *Malus domestica*^29^, *P. persica*^30^, *Rosa chinensis*^31^, *Fragaria vesca*^32^, *Rubus occidentalis*^33^, and *Solanum lycopersicum*^34^ (outgroup; not shown in the Figure). 'Cuiguan' pear and 'Suli' pear were closely clustered, indicative of a close genetic relationship. We also calculated the synonymous substitution rates (Ks) of collinear gene pairs using the 'Cuiguan' pear, apple, and peach genomes. The Ks density distribution revealed a peak shared by pear and apple (Supplementary Fig. S2), consistent with the recent whole-genome duplication event that occurred before the species differentiation between pear and apple occurred.

### Identification and analysis of *DAM* genes in the 'Cuiguan' and 'Suli' pear genomes

**Fig. 2:**
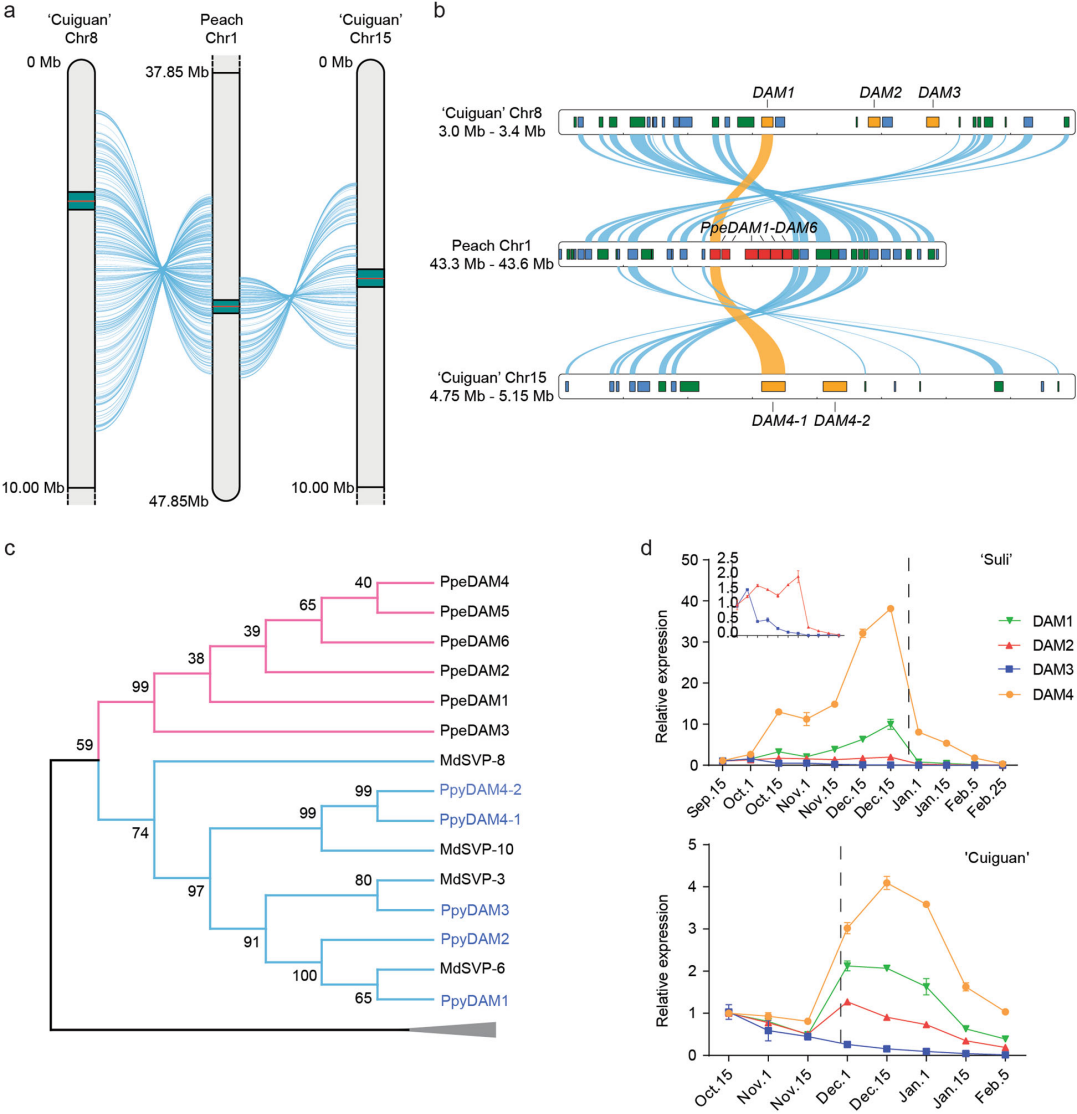
Genome-wide identification and expression analysis of *DAM* genes in pear. **a** Collinear blocks between peach chromosome 1 and 'Cuiguan' pear chromosomes 8 and 15. A 10 Mb partial chromosome region is shown around each collinear block. The detailed collinearity relationship of the green blocks is shown in (**b**). **b** Detailed collinear blocks between the peach *EVG* locus and pear *DAM* genes. Chromosomal segments are indicated by the horizontal bars. Genes in the positive and negative strands are shown in the green and blue boxes, respectively. The peach *DAM* genes are marked in red, and the pear *DAM* genes are marked in orange. The collinear *DAM* gene pairs are linked by orange curves, whereas the other genes are linked by blue curves. **c** Phylogenetic analysis of rosaceous DAM proteins. Ppe *Prunus persica*, Ppy *Pyrus pyrifolia*, Md *Malus domestica*. The pear *DAM* genes are shown in blue text. **d** *DAM* gene expression patterns in 'Suli' and 'Cuiguan' pears during a natural endodormancy cycle. The error bars indicate the standard deviations of three biological replicates. The dashed lines indicate the date of endodormancy release

*DAM* genes encode key bud endodormancy regulators in rosaceous plant species. Genome-wide analysis revealed five intact *DAM* genes in the 'Cuiguan' pear genome, which we renamed according to their chromosomal positions. Three and two *DAM* genes were tandemly arrayed on chromosomes 8 (*DAM1*–*3*) and 15, respectively. We named the two *DAM* genes on chromosome 15, which encode identical proteins, *DAM4-1* and *DAM4-2* (Fig. 2). Collinearity analysis revealed a strong collinear relationship between the peach *EVG* locus and loci on pear chromosomes 8 and 15 (Fig. 2a, b). The alignment of DAM proteins showed that the peach and pear DAM proteins are highly similar. However, the similarity between sequences from the same species was greater than those between different species (Supplementary Fig. S3). In the phylogenetic tree (Fig. 2c), the five pear DAMs clustered together with four apple SVP proteins and were grouped with six peach DAM proteins.

We next compared *DAM* gene expression patterns during the endodormancy cycle between 'Suli' pear and 'Cuiguan' pear (Fig. 2d). Because the *DAM4-1* and *DAM4-2* sequences are identical, their PCR products were indistinguishable. Consequently, we used *DAM4* to represent the expression of *DAM4-1* and *DAM4-2*. For 'Suli' pear, two expression patterns were observed for the four *DAM* genes. The *DAM3* expression level decreased continuously from September, when we started sampling, until endodormancy release. *DAM1*, *DAM2*, and *DAM4* expression levels were upregulated from mid-October, when endodormancy started, and peaked prior to endodormancy release, after which they decreased substantially. The *DAM3* expression pattern in 'Cuiguan' pear was similar to that in 'Suli' pear. *DAM1*, *DAM2*, and *DAM4* expression levels decreased shortly after endodormancy release, which was in contrast to the corresponding expression patterns in 'Suli’ pear.

### Establishment of VIGS and *DAM* gene silencing in pear buds

**Fig. 3:**
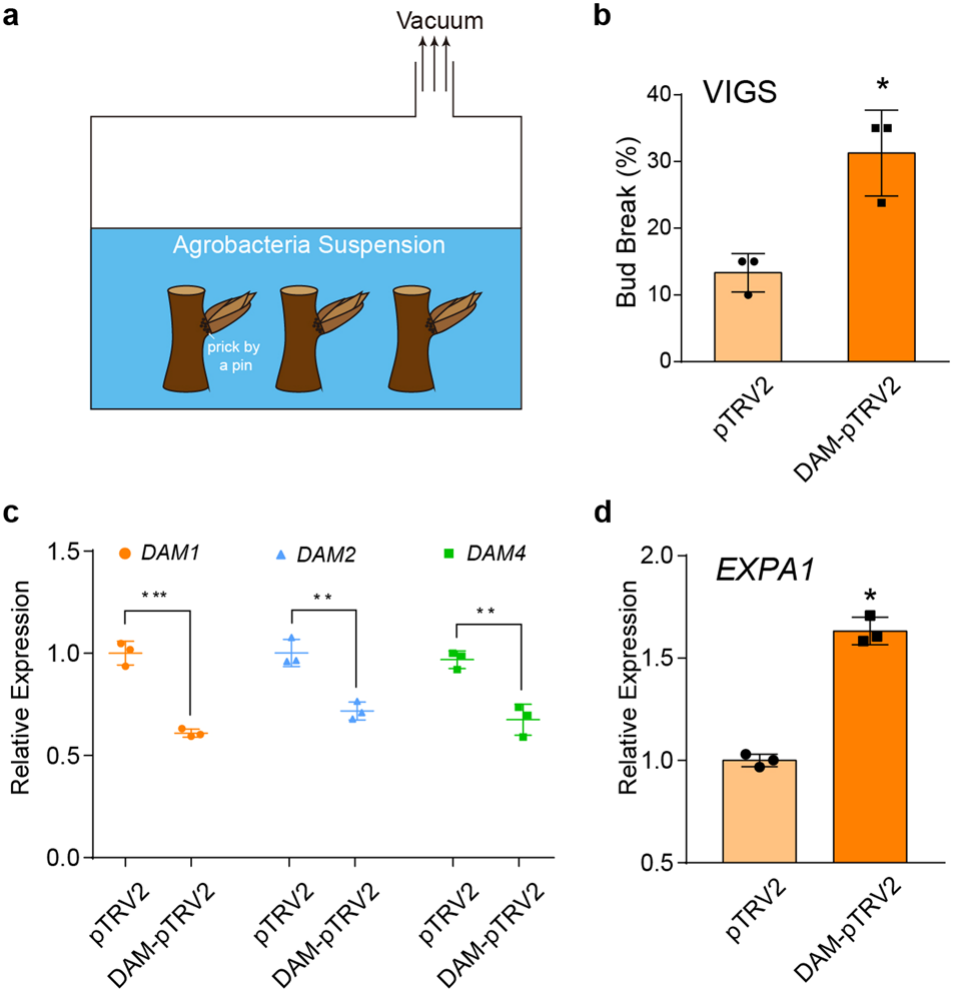
Virus-induced gene silencing of *DAM* genes in pear buds. **a** Agroinfiltration of cut pear shoots. **b** VIGS of pear *DAM* genes increased the floral bud break rate after 21 days of cultivation under forcing conditions. The error bars indicate the standard deviations of three biological replicates. The asterisk indicates a significant difference compared with the empty vector control according to Student’s *t*-test (*P < 0.05). **c** *DAM1*, *DAM2*, and *DAM4* expression levels in buds at 5 days after VIGS treatment. The error bars indicate the standard deviations of three biological replicates. The asterisks indicate significant differences compared with the empty vector control according to Student’s *t*-test (**P < 0.01; ***P < 0.001). **d** *EXPA1* expression in buds at 5 days after VIGS treatment. The error bars indicate the standard deviations of three biological replicates. The asterisk indicates a significant difference compared with the empty vector control according to Student’s *t*-test (*P < 0.05)

To further clarify DAM protein functions during the endodormancy cycle, we established a VIGS system in pear buds to study gene function during bud endodormancy. The experiment was conducted using excised 'Suli' pear shoots infected by Agrobacterium harboring pTRV1 and pTRV2-DAM (Fig. 3a). To silence *DAM* genes only, the selected fragment was taken from a relatively conserved region of *DAM* genes (half of the K-box domain) but did not include the conserved MADS-box region (Supplementary Fig. S4).

The bud break percentage was significantly higher in *DAM*-silenced dormant 'Suli' pear buds than in control buds after a 10-day forcing treatment (Fig. 3b), indicating that silencing *DAM* genes in pear buds promoted endodormancy release. At 5 days after agroinfiltration, quantitative real-time (qRT)-PCR analysis indicated that *DAM* gene (*DAM1*, *DAM2*, and *DAM4*) expression levels were significantly downregulated (Fig. 3c). Furthermore, the expression of *EXPANSIN A1* (*EXPA1*), which is related to endodormancy release^35^, was significantly upregulated after the *DAM* genes were silenced (Fig. 3d). The silencing of *DAM* genes may have promoted endodormancy release because of the associated induced expression of growth-related genes (e.g., *EXPA1*). Thus, we confirmed that high *DAM* gene expression levels are correlated with the maintenance of pear bud endodormancy.

### Artificial chilling accumulation and phytohormone contents

**Fig. 4:**
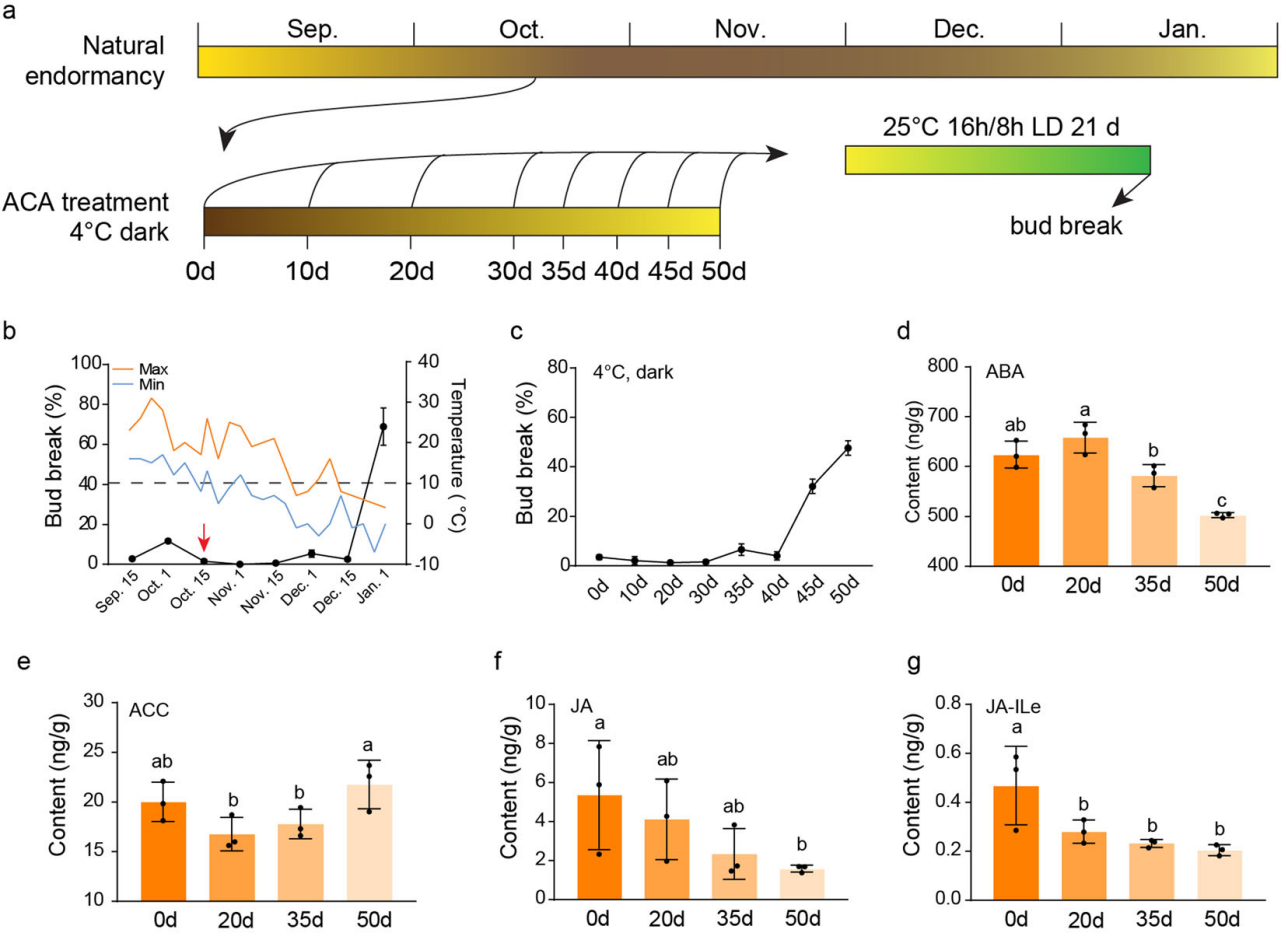
Endodormancy status and phytohormone contents in buds during ACA treatment. **a** Outline of ACA treatment and sample harvesting. **b** Endodormancy process (bud break percentage) of 'Suli' pear buds and daily temperature during the endodormancy transition. Orange and blue lines represent the maximum and minimum daily temperatures, respectively. The black dots indicate the bud break percentage on different sampling dates. The red arrow represents the sample used for ACA treatment. **c** Floral bud break rate during ACA treatment. **d**–**g** Changes in ABA, ACC, and JA contents in bud samples during ACA treatment. Phytohormone contents were measured by UPLC-MS/MS. The error bars indicate the standard deviations of three biological replicates, whereas the dots indicate individual values. The different letters above the bars indicate significant differences between samples according to one-way ANOVA (Duncan’s test, *P* < 0.05)

To determine the effects of long-term chilling on the regulation of endodormancy release, 1-year-old detached shoots of 'Suli' pear plants were collected on October 15, 2019, for ACA treatment (Fig. 4a). This date was selected because the 'Suli' pear buds were in the endodormancy stage and because the temperature was still greater than 10 °C (Fig. 4b). After a 30-day chilling accumulation period, the bud break percentage began to increase, exceeding 50% at 50 days, which is when bud endodormancy was released (Fig. 4c).

We also measured the abundance of several phytohormones in buds subjected to ACA treatment and those under natural conditions to confirm the effects of chilling accumulation. The ABA content was relatively high during the first 20 days of the ACA treatment, after which it decreased gradually, which was consistent with changes under natural conditions (Fig. 4d; Supplementary Fig. S5a). Interestingly, changes in the abundance of the ethylene precursor ACC were the opposite of those observed for the ABA content (Fig. 4e; Supplementary Fig. S5b), implying that ethylene influences endodormancy induction and release. Additionally, the jasmonate (JA and JA-Ile) contents tended to gradually decrease during the chilling accumulation period (Fig. 4f, g; Supplementary Fig. S5c).

### Screening of dormancy transition-related genes by RNA-seq

To characterize temporal gene expression changes that occur during the dormancy transition, we analyzed samples treated with ACA and those under natural conditions by RNA-seq. A total of 21 libraries from seven time points of the ACA treatment and 15 libraries from five time points of the natural dormancy cycle were sequenced. Sequencing generated ~252.16 Gb of clean reads, which we mapped to the previously released 'Suli' pear genome, with average mapping rates of 82.15% and 71.59% for the samples treated with ACA and those under natural conditions, respectively (Supplementary Fig. S6); however, when the new 'Cuiguan' pear genome was used as the reference sequence, the mapping rates were as high as 89.84% and 77.95%, respectively (Supplementary Fig. S6). Therefore, we analyzed the RNA-seq data based on the 'Cuiguan' pear genome sequence. The HISAT2 featureCounts-DESeq2 pipeline revealed 9392 differentially expressed genes (DEGs) in the ACA-treated samples and 14,673 DEGs in those under natural conditions (Supplementary Dataset).

Our analysis of the pear *DAM* gene expression patterns indicated that all the genes were differentially expressed when the results under the ACA treatment and natural conditions were compared. The *DAM1*, *DAM2*, and *DAM4* expression patterns were similar, with high expression levels during the early-ACA period (0–10 days) and decreasing levels toward endodormancy release (10–50 days, Supplementary Fig. S7a). In contrast, *DAM3* expression was continuously downregulated during the ACA period (Supplementary Fig. S7). These changes were in accordance with the expression patterns under natural conditions (Fig. 2c; Supplementary Fig. S7b). qRT-PCR analysis of functional *DAM* genes confirmed their expression patterns in the ACA-treated samples (Supplementary Fig. S7c).

**Fig. 5:**
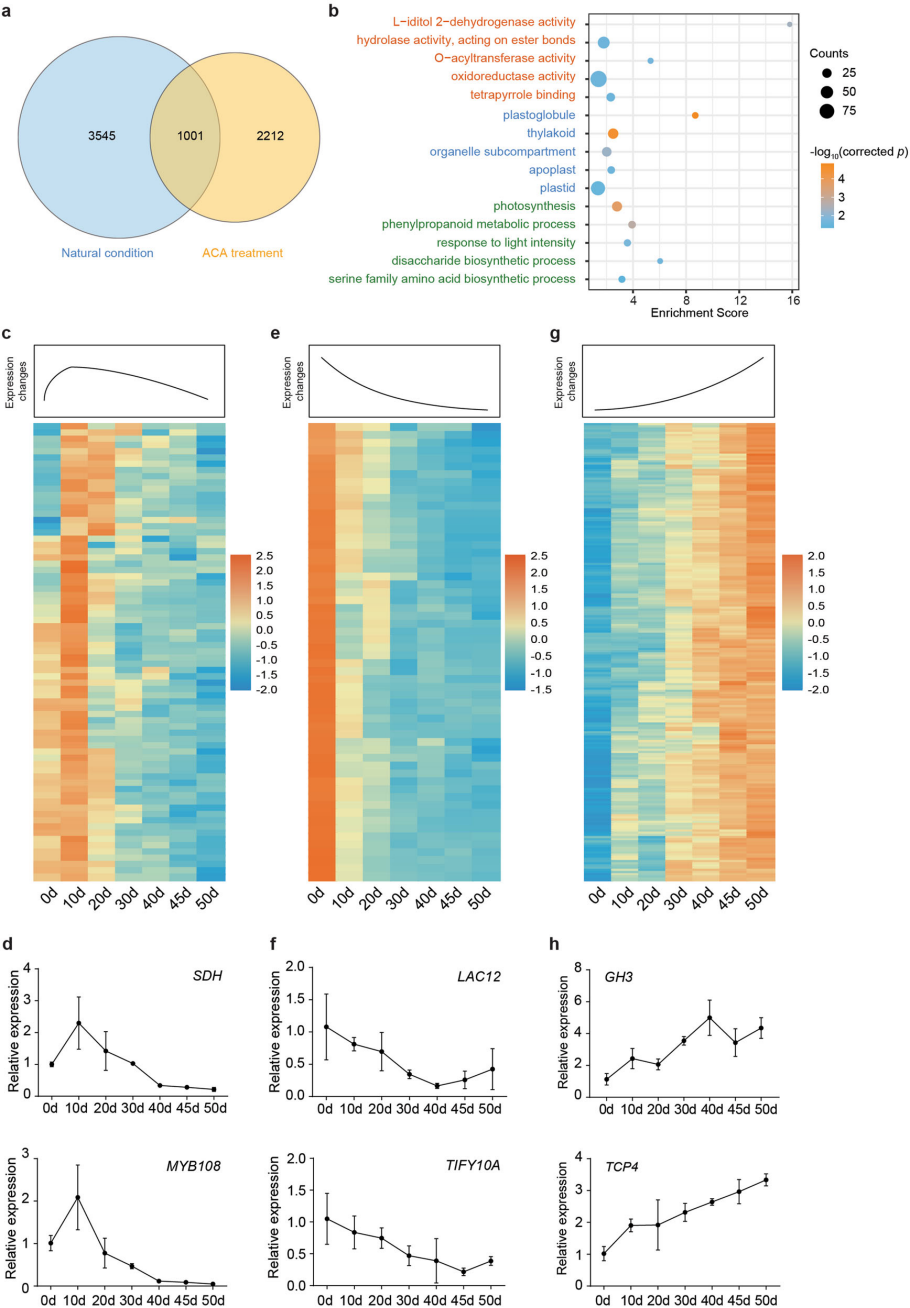
RNA-seq analysis of floral buds during ACA treatment. **a** Venn diagram of genes in specific clusters for ACA-treated samples and samples under natural conditions. The 1001 overlapping genes were considered candidate genes regulating endodormancy. The blue circle shows DEGs in samples under natural conditions (clusters 3, 5, 7, 8, and 9), whereas the yellow circle shows DEGs in ACA-treated samples (clusters 2, 4, 5, 6, and 9). **b** GO analysis of the 1001 putative candidate genes. The orange, blue, and green text correspond to the molecular function, cellular component, and biological process GO categories, respectively. The dot color represents the −log_10_ corrected *p* value (Benjamini–Hochberg corrected), whereas the dot size reflects the number of enriched genes. **c** Heatmaps of TPM values for ACA clusters 2/4 and (**d**) the expression of selected genes as determined by qRT-PCR. **e** Heatmaps of the TPM values for ACA cluster 5 and (**f**) the expression of selected genes as determined by qRT-PCR. **g** Heatmaps of the TPM values for ACA cluster 9 and (**h**) the expression of selected genes as determined by qRT-PCR. The line graphs in **c**, **e**, and **g** show the expression trends of the correlated clusters

To further identify the genes involved in the regulation of the dormancy transition, using Mfuzz analysis. we independently categorized the DEGs in ACA-treated samples and samples under natural conditions into clusters based on their expression patterns^36^. Nine clusters were obtained for each sample type (Supplementary Fig. S8). Clusters with linear changes during the ACA period (clusters 2, 4, 5, 6, and 9) as well as clusters 3, 5, 7, 8, and 9 for samples under natural conditions were further analyzed (Supplementary Fig. S8a, b). Specifically, the 1,001 overlapping genes between these two DEG pools were selected for further analyses (Fig. 5a).

GO analysis indicated that the 1001 genes were mainly associated with enzyme activities, plastoglobules, and phenylpropanoid metabolic processes (Fig. 5b), suggesting that these genes contribute to multiple biological activities. We analyzed the expression patterns of ACA-treated samples and identified genes in clusters 2/4 (ACA-clusters 2/4, Supplementary Table S3) and cluster 5 (ACA-cluster 5, Supplementary Table S4) with downregulated expression toward endodormancy release as candidate genes inhibiting endodormancy release. Cluster 9 genes for ACA-treated samples (ACA cluster 9, Supplementary Table S5) were considered candidate genes that promote endodormancy release.

Genes encoding transcription factors including NACs (NAC10, NAC40, and NAC88), MYB108, TCP12, ZAT12, ABRE-BINDING FACTOR 2 (ABF2), and DAM4 (Fig. 5c; Supplementary Table S3) and enzymes such as protein phosphatase 2C, sorbitol dehydrogenase, and E3 ubiquitin-protein ligase were identified in ACA clusters 2/4. The enriched GO terms among the DEGs in clusters 2/4 were porphyrin-containing compound metabolic process, tetrapyrrole metabolic process, and pigment metabolic process (Supplementary Fig. S9a). The expression levels of these genes were upregulated during the early-chilling accumulation period (0–20 days; October 15–December 15) and were downregulated toward endodormancy release (20–50 days; December 15–January 20) (Fig. 5c; Supplementary Fig. S9b). We further validated the expression patterns of these genes during the dormancy transition via qRT-PCR (Fig. 5d).

The expression of genes in ACA cluster 5 continuously decreased during the chilling accumulation period (Fig. 5e; Supplementary Fig. S10a). Several genes encoding transcription factors (e.g., ethylene response factor [ERF], basic helix–loop–helix [bHLH], and MADS-box types) were identified. Additionally, most pear *DAM* genes, including *DAM1*, *DAM2*, and *DAM3*, were categorized in this cluster. The expression of *DAM3* decreased more rapidly than did that of *DAM1* and *DAM2*. Moreover, the strigolactone esterase *DAD2* gene and several genes encoding antioxidant-related enzymes, such as glutathione S-transferase and peroxidase, were also included in this cluster. GO enrichment analysis suggested that the genes in this cluster participate in transcriptional regulation and protein dimerization (Supplementary Fig. S10b). The expression profiles of selected genes in the samples under natural conditions were similar to those in the ACA-treated samples (Fig. 5f).

In ACA cluster 9, the expression levels of genes encoding transcription factors (e.g., bHLH, TCP, ERF, G-box-binding factor, auxin response factor, and zinc finger protein types) increased throughout the chilling accumulation period (Fig. 5g). Additionally, the expression levels of some structural genes, including *YUCCA*, *cyclin-J18* (*CYCJ18*), fructokinase, and sucrose synthase genes, were significantly upregulated during the chilling accumulation period (Fig. 5g; Supplementary Fig. S11a; Supplementary Table S5). Interestingly, the expression of some genes related to epigenetic regulation, including those encoding histone H2AX, methyltransferase, histone-lysine N-methyltransferase SUVR2-like, and acyltransferase, was also upregulated during prolonged chilling accumulation (Supplementary Table S5), suggesting that epigenetic modifications might be involved in chilling-induced endodormancy release. The enriched GO terms among the DEGs in cluster 9 were pyrimidine-containing compound process, ribose phosphate biosynthetic process, RNA processing, and reproductive system development (Supplementary Fig. S11b). In addition, the expression levels of candidate genes [*TCP4* and *GH3* (encoding glucan endo-1,3-beta-glucosidase 3)] were upregulated during the chilling accumulation period (Fig. 5h), as confirmed via qRT-PCR.

### H3K4me3 chromatin immunoprecipitation and sequencing (ChIP-seq) analysis of dormant 'Suli' pear buds during the chilling accumulation period

To investigate the possible epigenetic regulation of endodormancy release, we determined the genome-wide distribution of H3K4me3 modifications in dormant pear buds. The ChIP-seq data revealed that H3K4me3 histone marks were mainly associated with transcription start sites (TSSs) (Supplementary Fig. S12a) in all ACA-treated samples. Moreover, gene expression was closely related to the presence of H3K4me3 at the TSS (Supplementary Fig. S12b–f).

**Fig. 6:**
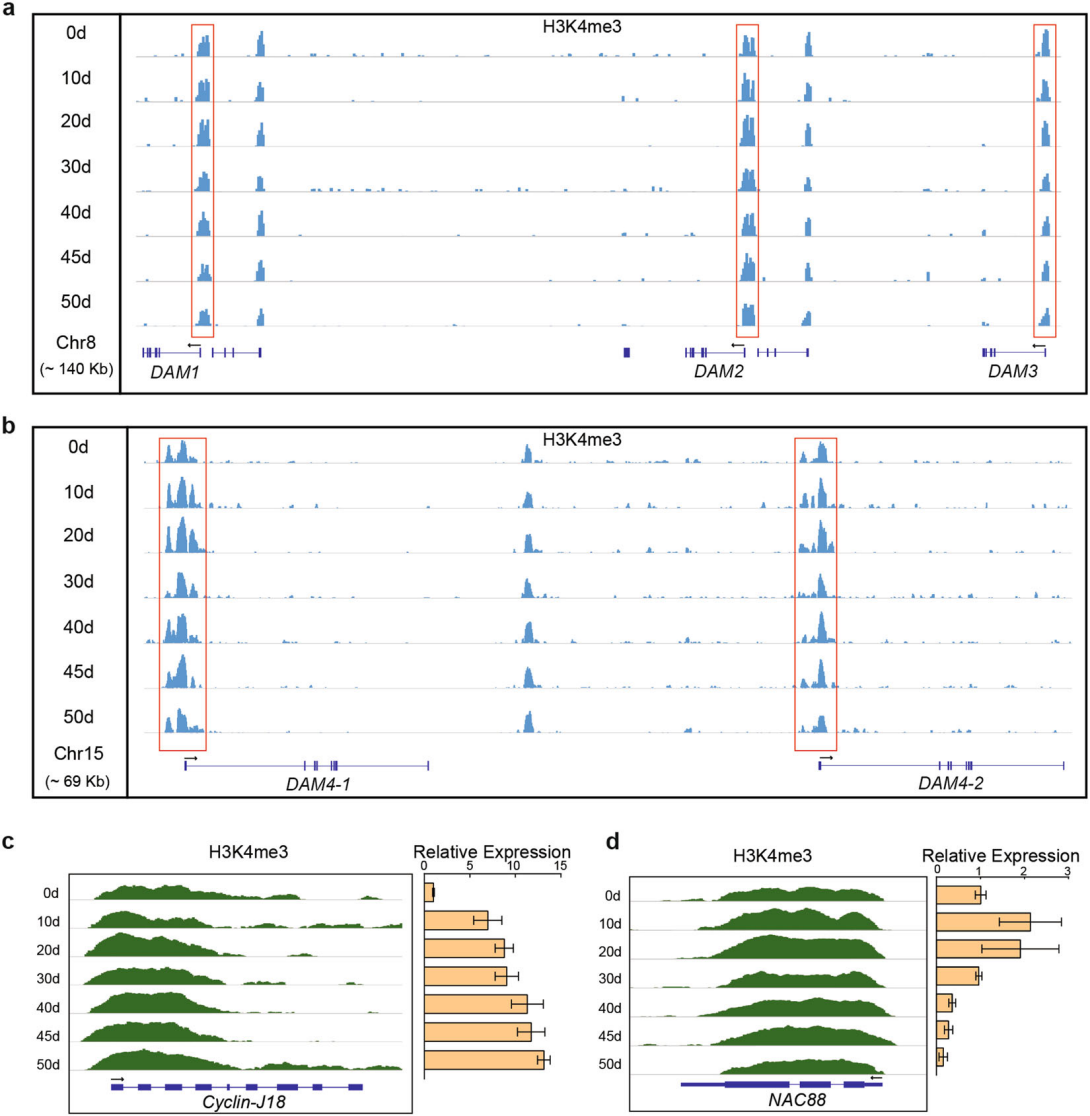
ChIP-seq analysis of candidate genes for endodormancy release. **a**, **b** Enrichment of H3K4me3 in *DAM* loci on 'Cuiguan' pear chromosomes 8 and 15. The peaks represent the normalized H3K4me3 signals. The exons are indicated by blue bars, whereas the introns are indicated by gray horizontal lines. The transcription orientation is marked above the gene structures. **c** H3K4me3 level at the *CYCJ18* (EVM0000777.1) locus. The arrow indicates the coding direction. The exons are indicated by blue boxes, whereas the introns are indicated by lines. The yellow bars represent the relative expression of *CYCJ18* during ACA treatment. The error bars indicate the standard deviations of three biological replicates. **d** H3K4me3 level at the *NAC88* (EVM0012159.1) locus. The arrow indicates the coding direction. The exons are indicated by blue boxes, whereas the introns are indicated by lines. The yellow bars represent the relative expression of *NAC88* during ACA treatment

During the ACA period, we found that the expression of *DAM* genes in ACA clusters 4 and 5 decreased toward endodormancy release (Supplementary Fig. S7; Supplementary Tables S3, S4). We also showed that H3K4me3 modification was clearly enriched around the TSS of all *DAM* genes. For *DAM1* and *DAM3*, the enrichment of H3K4me3 decreased beginning at the middle of the ACA period and was lowest after 50 days (Fig. 6a). However, for *DAM4-1* and *DAM4-2*, H3K4me3 enrichment increased soon after initiating ACA treatment and then decreased until the end of the treatment period (Fig. 6b).

Finally, our RNA-seq data indicated that the expression of a cell division-related gene, *CYCJ18* (cluster 9), of the cyclin family steadily increased during the chilling accumulation period (Supplementary Table S5). The ChIP-seq data revealed that H3K4me3 was enriched at the *CYCJ18* locus during the chilling accumulation period (Fig. 6c). Additionally, the expression of the cold-induced transcription factor-encoding gene *NAC88*, whose H3K4me3 level increased and then decreased during the chilling accumulation period, was upregulated during endodormancy but downregulated during endodormancy release (Fig. 6d).

## Discussion

### The 'Cuiguan' pear genome is a new reference genome for cultivated asian pear

PacBio long reads can enhance the accuracy and integrity of genome assemblies, whereas Hi-C sequencing enables pseudochromosomes to be correctly assembled. Compared with the 'Suli' pear (*P. bretschneideri*) genome sequence released by Wu et al.^23^, the 'Cuiguan' pear (*P. pyrifolia*) genome sequence constructed in this study had a higher contig N50 value. The LAI represents genome assembly continuity because LTR retrotransposons are poorly assembled in draft genomes. In the current study, the LAI of the 'Cuiguan' pear genome was 17.62 (Table 1), which is similar to that of the *Arabidopsis thaliana* TAIR10 reference genome^37^, while BUSCO evaluation confirmed that the 'Cuiguan' pear genome was relatively complete. Moreover, its RNA-seq read mapping rate was relatively high (Supplementary Fig. S6). These results imply that this is a high-quality reference genome that could be useful for genome-based research on Asian pear. 'Cuiguan' and 'Suli' are the main pear cultivars grown in China and are usually considered to be *P. pyrifolia* and *P. bretschneideri*, respectively. In this study, they were clustered together, revealing a very close genetic relationship (Fig. 1b) and supporting our earlier conclusion that Chinese white pear cultivars such as 'Suli' should be treated as *P. pyrifolia* cultivars (i.e., the *P. pyrifolia* white pear group)^38–40^.

### Characterization and function of pear *DAM* genes

Three MADS-box proteins (DAM1/2/3 or MADS13-1/-2/-3) from 'Suli' and 'Kosui' pear cultivars were previously identified in studies involving homology-based cloning^9–11^. Only one intact *DAM* gene and two partial *DAM* genes have been annotated in the released 'Suli' pear genome^10^. To unify the naming of pear *DAM* genes, the five *DAM* genes detected in the 'Cuiguan' pear genome were renamed according to their chromosomal locations (Supplementary Table S6). Phylogenetic analysis (Fig. 2) implied that apple and pear *DAM* genes evolved before the separation of *Malus* and *Pyrus* and that they share the same origin as *Prunus DAM* genes. Specifically, peach *DAM* genes were tandemly arrayed at the *EVG* locus^5^, and pear *DAM1/2/3* were tandemly arrayed on chromosome 8. We also detected a strong collinear relationship between the *DAM* locus on peach chromosome 1 and that on pear chromosome 8 (Fig. 2a). However, peach and pear *DAM* genes clustered separately in the phylogenetic tree, suggesting that these clusters were derived from segmental duplication events that occurred after the separation of the tribes Amygdaleae and Maleae.

A previous study proved that the regulated *DAM1*, *DAM2*, and *DAM4* (originally named *MADS13-1*, *MADS13-2*, and *MADS13-3*, respectively) expression patterns in 'Kosui' Japanese pear leaf buds are consistent with endodormancy establishment and release^9^. In the present study, we identified *DAM3* and *DAM4-2* as additional pear *DAM* genes. Two expression patterns were detected for these *DAM* genes, especially in 'Suli' pear, which has a high CR. *DAM1*, *DAM2*, and *DAM4* expression levels increased during endodormancy but decreased rapidly after entering the ecodormancy stage, consistent with the results of our previous studies^10,13^ and with reported *DAM6* expression patterns in peach and Japanese apricot^6,41^. In contrast, *DAM3* expression decreased continuously beginning in September, reaching its lowest level upon endodormancy release. Although the *DAM3* expression pattern was similar in 'Cuiguan' pear and 'Suli' pear, the expression level was much lower and decreased earlier in 'Cuiguan' pear. Moreover, *DAM1*, *DAM2*, and *DAM4* expression changed more in 'Suli' pear than in 'Cuiguan' pear. Because endodormancy maintenance requires high *DAM* gene expression levels, our results suggest that the limited changes in *DAM1*, *DAM2*, and *DAM4* expression during endodormancy might be responsible for the relatively weak dormancy of 'Cuiguan' pear. Previous studies also concluded that differences in the expression of these *DAM* genes might be related to CR diversity^9,13^.

Ectopic expression of *PmDAM6* in poplar reportedly inhibits plant growth and delays bud break^6^. Overexpression of *PpyDAM4* (previously designated *PpyDAM3*) in pear calli was also shown to inhibit growth and downregulate *CYCLIN D* and *EXPASIN 1* expression^13^. Except for the peach *evg* mutant^5^, there has been no direct genetic evidence of DAM functions related to the floral bud endodormancy of Amygdaloideae plant species, likely because of a lack of effective transformation techniques and a long juvenile period. In this study, we established a VIGS technique to functionally characterize genes in pear floral buds. Previous investigations have confirmed that tobacco rattle virus (TRV) can infect apple and pear^42–44^. Therefore, TRV was used to silence *DAM* genes in dormant pear buds to further clarify DAM functions during bud dormancy transition. Because we were unable to identify the buds in which *DAM* genes were silenced, we collected all the buds. Our subsequent analysis confirmed that the silencing, or at least downregulation, of *DAM1*, *DAM2*, and *DAM4* expression led to an increase in bud break percentage under forcing conditions (Fig. 3). As expected, the expression of the downstream gene *EXPA1* was significantly upregulated in pear buds when *DAM1*, *DAM2*, and *DAM4* were silenced. Thus, our results confirmed that *EXPA1*, which is closely associated with bud break, is located downstream of the *DAM* genes in pear buds. Future studies involving the overexpression of these genes in transgenic plants may help elucidate the functions of encoded proteins during the dormancy transition.

### Candidate genes potentially regulating pear endodormancy release

Analyses of RNA-seq data for buds collected under natural conditions and those of plants treated with specific chemicals have revealed many candidate genes encoding key dormancy regulators^3,17,43–45^, but the precise functions of these genes remain unclear. However, under natural conditions, bud status can be affected by disease, insect pests, and other dynamic environmental cues, which might affect the identification of candidate genes. ACA treatment may enable a more accurate gene expression analysis. Gabay et al.^45^ recently used ACA-treated vegetative buds to quantify the metabolite contents in European pear, revealing that fatty acids including α-linolenic acid influence the dormancy transition. In the present study, we comprehensively analyzed the RNA-seq data for ACA-treated floral buds and the floral buds under natural conditions.

Mfuzz analysis allowed us to cluster DEGs in ACA-treated samples and samples under natural conditions into nine groups. Because *DAM1*, *DAM2*, and *DAM3* were grouped in ACA cluster 5 and encode proteins that usually form functional dimers, the transcription factors-encoding genes (e.g., *ZAT10*, *RAP2.3*, *TIFY10A*, and *ERF5*) in cluster 5 might be closely related to *DAM* genes with respect to expression and function. GO analysis indicated that ACA cluster 9 genes are associated with reproduction-related processes and cell division (Supplementary Fig. S11), implying that cell division is activated and floral organs develop during the progression of chilling accumulation in preparation for endodormancy release and flowering. This finding is consistent with our recent findings^35^. Among these genes, *CYCJ18* is responsible for nuclear division^46^, whereas *GH3*, belonging to the glucan hydrolase superfamily, encodes an enzyme that hydrolyzes 1,3-β-glucan, which regulates dormancy in poplar^15,47^. *GH3* expression was upregulated during the chilling accumulation period, similar to the expression of *GH17* members in poplar^47^, suggesting that *GH3* might be crucial for callose degradation in pear buds and the promotion of endodormancy release. However, the functions of these genes need to be thoroughly characterized in future studies.

Among the transcription factor-encoding genes, *NAC10*, *NAC40*, and *NAC88* expression levels were continuously downregulated toward endodormancy release, similar to *DAM* gene expression (Figs. 5, 6; Supplementary Fig. S9b). Earlier research indicated that NAC10 and NAC88 may be involved in plant responses to low temperature as well as the regulation of dormancy transition^48^. Any potential relationships among *NAC10*, *NAC88*, and *DAM4* need to be experimentally verified, as do the functions of NAC10 and NAC88 related to bud dormancy. The expression of several genes encoding bHLH transcription factors (e.g., bHLH10-like, bHLH90, and bHLH91-like) was also upregulated during the chilling accumulation period, which is indicative of a potential role in endodormancy release (Fig. 5g). The expression of an identified TCP family gene, *TCP4*, was upregulated during the chilling accumulation period, with peak levels at endodormancy release. Interestingly, in *A. thaliana*, TCP family proteins interact with the bHLH protein PIF4, target the gibberellin (GA) biosynthesis-related gene *GA20ox1* and the atypical HLH transcription factor-encoding gene *PRE6*, thereby regulating thermomorphogenic growth^49^. The expression of *PRE1*, which positively regulates GA signaling and cell elongation in *A. thaliana*^50^, was also upregulated in pear buds during the chilling accumulation period. Thus, TCP4 may interact with bHLH proteins to activate *PRE1*, *GA20OX2*, and other cell division- and elongation-related genes (e.g., *CYCLIN* and *EXPANSIN*) to induce endodormancy release.

### ChIP-seq analysis reveals a possible association of H3K4me3 with dormancy in pear

Earlier research indicated that H3K4me3, which is a histone marker for active transcription, is involved in dormancy regulation in peach, sweet cherry, chestnut, and kiwifruit^16–18,51^. Our current data show that H3K4me3 levels are correlated with the expression of specific genes (Fig. 6) during the dormancy cycle. Additionally, these findings support the fact that pear *DAM* genes are targeted for H3K4me3 modifications during the chilling accumulation period (Fig. 6a, b), which is consistent with the results of related studies on peach, sweet cherry, and kiwifruit^16,18,51^. Although it is unclear how chilling regulates histone modifications at *DAM* loci, recent research has revealed the presence of small RNAs and noncoding RNAs at peach *DAM* loci, implying that they contribute to the regulation of histone modifications at these sites^19^.

We also observed that the gene expression of *NAC88* and *CYCJ18* was correlated with H3K4me3 modification (Fig. 6) during the dormancy transition. In poplar trees exposed to drought stress, the expression of genes encoding NAC transcription factors is reportedly controlled by H3K9ac histone modifications mediated by AREB1^52^, while *CYCJ18* affects nuclear division cycles during early *Drosophila* embryogenesis^46^. In previous investigations, we detected dividing cells as well as an increase in the number of nuclei in buds during maintenance and release of endodormancy^35^. Moreover, in poplar, SVL inhibited *cyclin-D* through *EARLY BUD BREAK 3* (*EBB3*) expression, which is epigenetically regulated by H3K27me3 to inhibit endodormancy release and bud break^53^. Our results suggest that cyclin genes are direct targets for histone modifications during endodormancy release. Indeed, taken together, these observations suggest that H3K4me3 modification is associated with the regulation of endodormancy release and that it does not target only *DAM* genes.

In conclusion, we assembled the genome of the sand pear cultivar 'Cuiguan', which should prove useful as a reference genome for future molecular studies. Using this new reference genome, we identified five *DAM* genes tandemly arrayed on chromosomes 8 and 15 and unified the names of the *DAM* genes according to their chromosomal positions. We established a VIGS technique applicable to pear floral buds, thereby providing researchers with a new method for functionally characterizing genes during the dormancy cycle of floral buds, especially those from fruit trees with long juvenile periods. This new reference genome enables higher-quality RNA-seq and ChIP-seq analyses that can be used in the future to identify additional candidate genes that may be involved in endodormancy release.

## Materials and methods

### Plant materials and the ACA

The leading Chinese sand pear (*P. pyrifolia*) cultivar 'Cuiguan' grown in China was used for genome sequencing. Dormancy regulation was investigated using 'Suli' pear (*P. pyrifolia* White Pear Group; also named *P. bretschneideri*) samples with known dormancy transition phenotypes and an available sequenced genome.

For samples under natural conditions, shoots were collected starting on September 15, 2016, and 2017, as previously described^13,54^. ACA treatment was completed using detached branches from 1-year-old shoots to eliminate the effects of the external environment. Shoots were collected starting on October 15, 2019. 'Suli' pear buds entered the endodormancy stage at this time point. The shoots were randomly divided into three groups to form three biological replicates and incubated at 15–18 °C for 3 days. Afterward, they were kept in a phytotron at 4 °C (with 90% humidity) for ACA treatment until endodormancy release (up to 50 days). Lateral flower buds were removed from the detached shoots at 0, 10, 20, 30, 35, 40, 45, and 50 days after initiating the ACA treatment, immediately frozen in liquid nitrogen, and stored at −80 °C until analysis. The dormancy status at each time point was determined by calculating the bud break percentage under forcing conditions (25 °C/23 °C, 16-h day/8-h night cycle) for 21 days.

### Genome assembly

Approximately 62.45 Gb of PacBio subreads with a mean length of 12,466 bp were generated for genome assembly. We first used Canu^55^ to correct the PacBio reads. Canu and WTDBG (https://github.com/ruanjue/wtdbg) were used to independently assemble the high-quality PacBio reads, and five rounds of polishing were carried out by Pilon (https://github.com/broadinstitute/pilon) using Illumina short reads. Self-alignment was performed to remove redundancies with MUMmer^56^. The Canu and WTDBG draft assemblies were merged using Quickmerge (https://github.com/mahulchak/quickmerge), and Pilon was used to polish the merged assembly. We generated 74.03 Gb of clean data from the Hi-C library for sequence assembly at the chromosome level. BWA^57^ was used to align reads to the assembly, and only uniquely mapped read pairs were retained. Additionally, invalid read pairs were filtered with HiC-Pro (v2.8.1)^58^, and valid read pairs that were used for subsequent analysis accounted for 81.33% of the uniquely mapped read pairs. LACHESIS software^59^ was used to cluster, order, and orient the genome sequences into pseudochromosomes with the parameters CLUSTER_MIN_RE_SITES=43, CLUSTER_MAX_LINK_DENSITY=2, CLUSTER_NONINFORMATIVE_RATIO=2, ORDER_MIN_N_RES_IN_TRUN=25, and ORDER_MIN_N_RES_IN_SHREDS=21.

### Genome annotation

Repeat sequences were predicted as follows. A de novo repeat library was constructed with LTR FINDER (v1.05)^60^, MITE-Hunter^61^, RepeatScout (v1.0.5)^62^, and PILER-DF (v2.4)^63^ using the default parameters. The database was then classified using PASTEClassifier^64^ and merged with Repbase as the final repetitive sequence database. Finally, RepeatMasker (http://www.repeatmasker.org/) was used to screen any repeats. The LAI was independently calculated using the LTR_retriever pipeline (https://github.com/oushujun/LTR_retriever).

The EVM (v1.1.1) pipeline^65^ was used to predict genes in the assembled genome sequence. Briefly, the default parameters of GenScan^66^, Augustus (v2.4)^67^, Glimmer HMM (v3.0.4)^68^, Gene ID (v1.4)^69^, and SNAP (v2006-07-28)^70^ were applied for de novo gene prediction. The default parameters of GeMoMa (v1.3.1)^71^ were used to predict homologs in the *A. thaliana*, *F. vesca*, and *P. bretschneideri* databases. The PASA program (v2.0.2)^72^ was used to predict unigene sequences based on the RNA-seq data for 'Cuiguan' pear. All the acquired data were then combined and revised using EVM and PASA. Protein-coding genes were annotated based on BLAST searches (e-value cutoff of 1e^−5^) of the information housed in the NR (http://www.ncbi.nlm.nih.gov), KOG (ftp://ftp.ncbi.nih.gov/pub/COG/KOG), GO (http://geneontology.org/), KEGG (http://www.genome.jp/kegg), and TrEMBL (http://www.UniProt.org/) databases.

### Phytohormone quantification

We entrusted Shanghai Applied Protein Technology Co., Ltd. (APTBIO, Shanghai, China), to extract and quantify the phytohormones in pear buds as previously described^73^.

### RNA extraction and RNA-seq

Total RNA was extracted from buds using a modified cetyltrimethylammonium bromide method^74^. We entrusted Novogene Co., Ltd. (Beijing, China), and BGI (Shenzhen, China) to construct and sequence the RNA-seq library of ACA-treated samples and samples under natural conditions, respectively. The raw data were processed during a quality control step to produce clean data.

### cDNA synthesis and qRT-PCR

First-strand cDNA was synthesized from 1 μg of total RNA using HiScript II Q RT SuperMix for qPCR (+gDNA wiper) (Vazyme Biotech Co., Ltd., Nanjing, China). The cDNA was subjected to a qRT-PCR assay, which was completed with iTaq Universal SYBR Green SuperMix (Bio-Rad Laboratories, Beijing, China), as previously described^13^. The pear actin (JN684184) gene was used as a reference control because it is constitutively expressed during the dormancy transition^21^. The qRT-PCR primers used are listed in Supplementary Table S8.

### ChIP-seq

The modified ChIP method used in this study was based on protocols described by Haring et al.^75^ and Li et al.^76^. Scales were removed from buds collected at 0, 10, 20, 30, 40, 45, and 50 days after the ACA treatment was started. The buds were treated with 1% (w/v) formaldehyde for 10 min under vacuum at room temperature in the cross-linking step. Afterward, 1.25 M glycine was added, and the tissues were incubated under vacuum for 5 min to terminate the cross-linking reaction. The supernatant was discarded, and the tissues were washed twice with distilled water and then allowed to dry on filter paper. The tissues were then frozen in liquid nitrogen and stored at −80 °C until analysis. After extracting the nuclei, 320 μl of nuclear lysis buffer [50 mM Tris-HCl (pH 8.0), 10 mM ethylenediaminetetraacetic acid, 1% (w/v) sodium dodecyl sulfate, 1 mM phenylmethylsulfonyl fluoride, and proteinase inhibitor cocktail] was added. The chromatin solution was subsequently sonicated 30 times with a bioruptor (30 s each time at 30 s intervals). The solution was centrifuged (16,000 × *g* at 4 °C for 5 min), after which 300 μl of the supernatant was transferred to a new 2-ml tube. Afterward, 1660 μl of ice-cold ChIP Ab incubation buffer was added to the chromatin solution, which was thoroughly mixed. A 60 μl aliquot was used as the input. ChIP-grade anti-H3K4me3 antibodies (Abcam, Cambridge, UK) and Pierce ChIP-grade Protein A/G magnetic beads (Thermo Fisher Scientific, Waltham, MA, USA) were used for IP. Following a reverse cross-linking step, the chromatin was purified with an Easy Gel Extraction & Clean-up Kit (Zhejiang Easydo Biotechnology Co., Ltd., Hangzhou, China) and eluted with ddH_2_O for sequencing. After immunoprecipitation of the samples (three biological replicates), the precipitated DNA was combined for deep sequencing analysis. We entrusted Novogene Co., Ltd., to construct and sequence the ChIP-seq library.

### Phylogenetic, RNA-seq, and ChIP-seq analyses

Detailed procedures of the phylogenetic, RNA-seq, and ChIP-seq analyses are described in Methods S1, Methods S2, and Methods S3.

### VIGS assay of pear buds

A VIGS assay was completed with TRV. A 244-bp reverse complementary fragment (end of the K-box) of *DAM4* was cloned and inserted into a pTRV2 vector using the primers listed in Supplementary Table S9. The construct was then inserted into Agrobacterium EHA105 cells. pTRV1 and pTRV2 empty vectors were inserted into separate EHA105 cells, and pTRV2 served as a negative control. The VIGS assay was performed using 'Suli' pear buds. After the bacteria reached the logarithmic growth phase, the culture was diluted with infection buffer [10 mM MgCl_2_, 10 mM MES (pH 5.6), and 100 μM acetosyringone] until an OD_600_ of 0.8 was reached. Equal volumes of pTRV1 and pTRV2 bacterial solutions were combined and incubated at room temperature for 1 h. 'Suli' shoots were collected on November 1, 2019 (with buds in the endodormancy stage), from plants growing under natural conditions and then cut into individual nodes. Syringe needles were used to pierce the bud and branch junction. The single-node shoots were subsequently soaked in the bacterial solution for 10 min at room temperature conditions under vacuum. The shoots were then placed on water-soaked floral foam and incubated at 25 °C/23 °C (light/dark) for 10 days. Bud samples were collected 5 days after infection, immediately frozen in liquid nitrogen, and stored at −80 °C until analysis. Bud break percentages were calculated at 10 days after infection when the bud scales were open and green tips were visible.

## Supplementary information

Supplementary_figures

Supplementary experimental procedures

Supplementary_tables

Supplementary dataset

## Data Availability

The accession numbers of the genes in this work are listed in Supplementary Tables S3, S4, S5, and S7. The RNA-seq and ChIP-seq data for bud dormancy have been deposited in the NCBI database under BioProject number PRJNA669907. The whole-genome sequence data reported in this paper have been deposited in the Genome Warehouse at the National Genomics Data Center, Beijing Institute of Genomics (China National Center for Bioinformation), Chinese Academy of Sciences, under accession number GWHBAOS00000000 and are publicly accessible at https://bigd.big.ac.cn/gwh.
