# Supplementary material for: High-quality genome assembly of 'Cuiguan' pear (Pyrus pyrifolia) as a reference genome for identifying regulatory genes and epigenetic modifications responsible for bud dormancy
Source: Hortic Res. 2021 Sep 1;8:197. doi: 10.1038/s41438-021-00632-w (PMC8408243; doi:10.1038/s41438-021-00632-w)
Supplement: Supplementary file 1 — Supplementary_figures [file 41438_2021_632_MOESM1_ESM.docx]

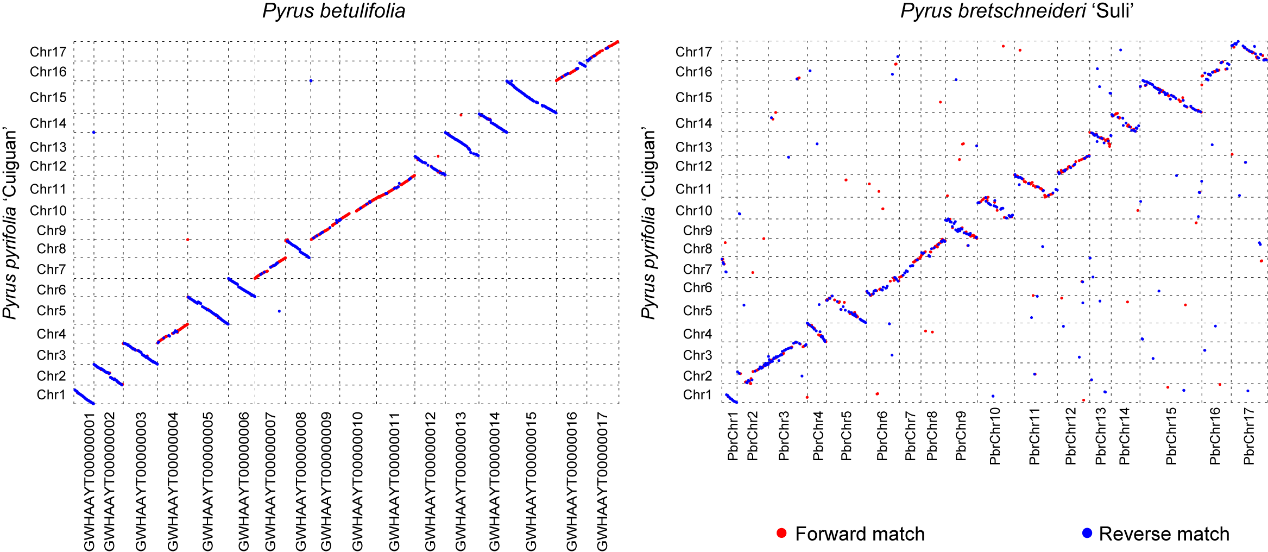


Figure S1 Chromosome level alignment between ‘Cuiguan’ and *P. betulifolia* (a), or between ‘Cuiguan’ and ‘Suli’ (b). Each dot indicates genomic sequences (over 25 Kb) that are matched with the similarity over 90%. The forward matches are indicated with red dots, while the reverse-complement matches are indicated with blue dots. MUMmer software package was used to conduct, filter and plot the alignments.


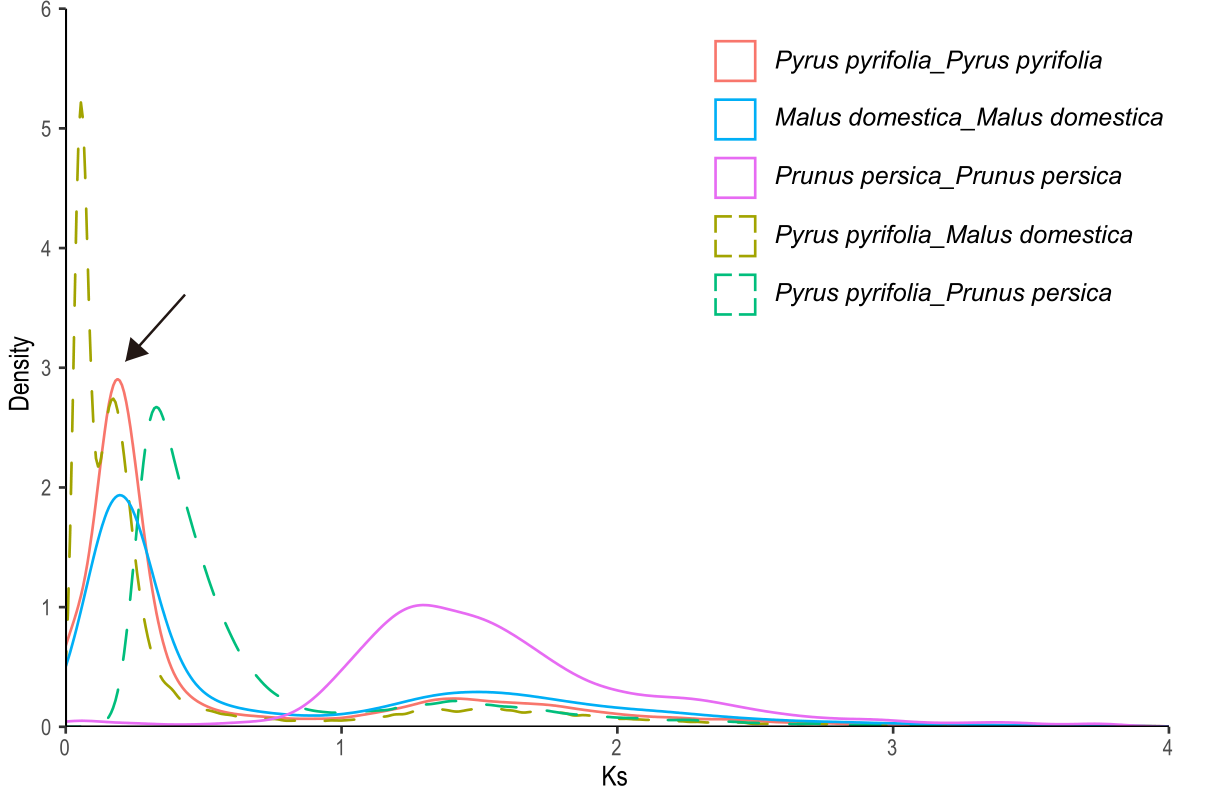


Figure S2. Distribution of synonymous substitutions rates (Ks) for collinear gene pairs among ‘Cuiguan’, apple and peach. The arrow indicates a genome duplication event shared by pear and apple.


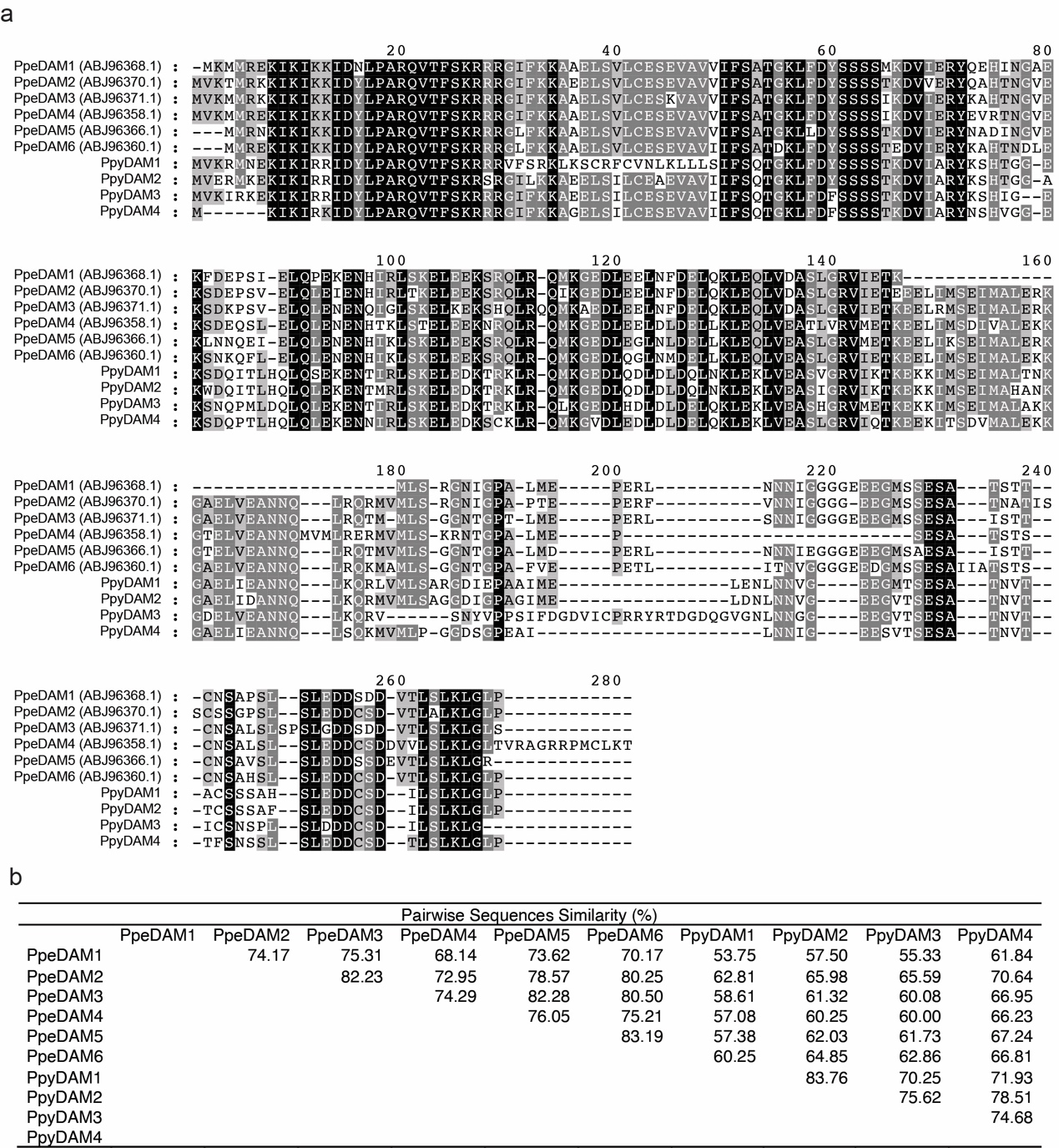


Figure S3. The sequence alignment and pairwise sequence similarity of pear and pear DAM proteins. Ppe. *Prunus persica*; Ppy. *Pyrus pyrifolia*.


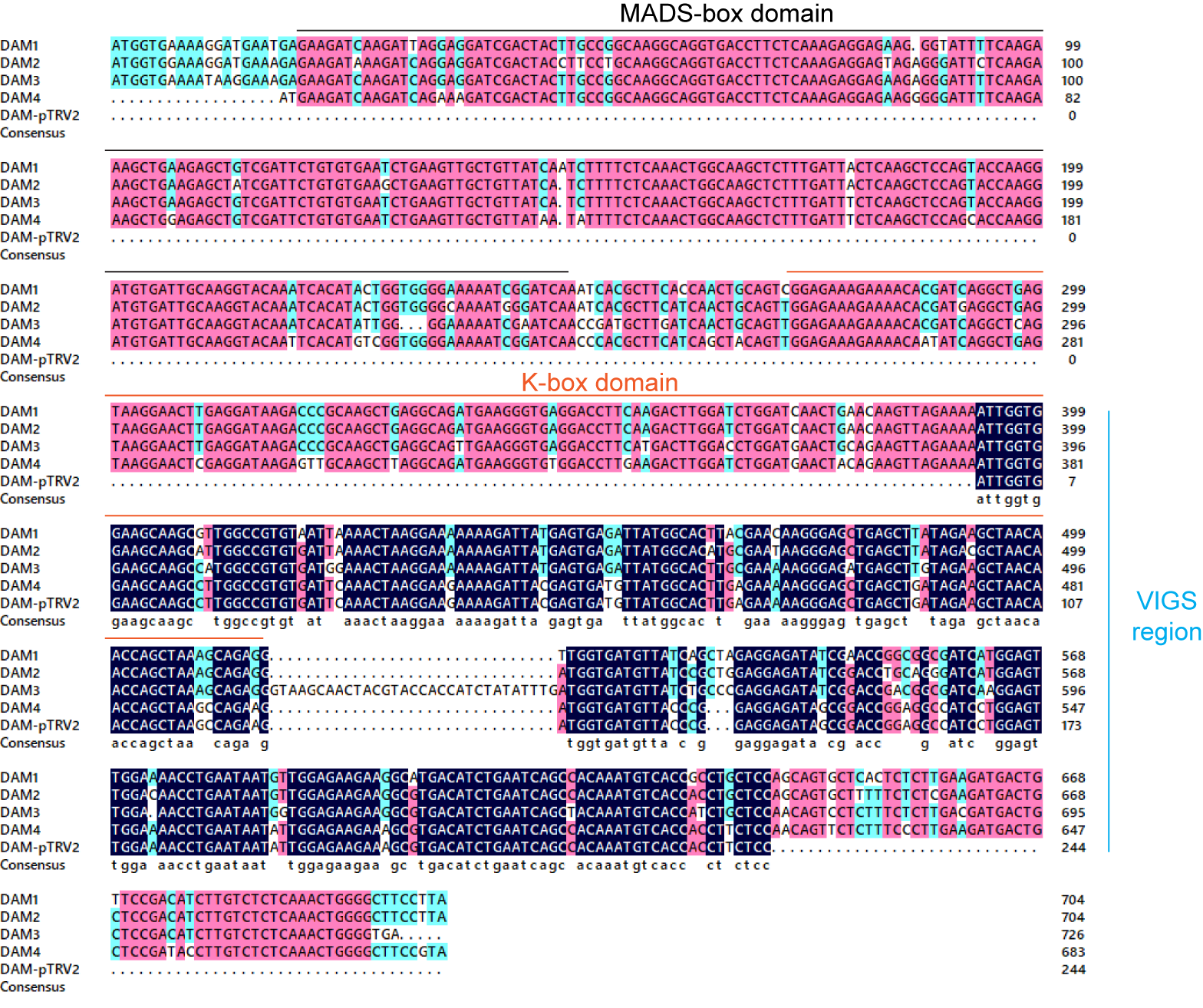


Figure S4 Alignment of *DAM* sequences and VIGS region. The black line indicates MADS-box domain while the orange line indicates K-box domain. Dark blue background presents the VIGS region.


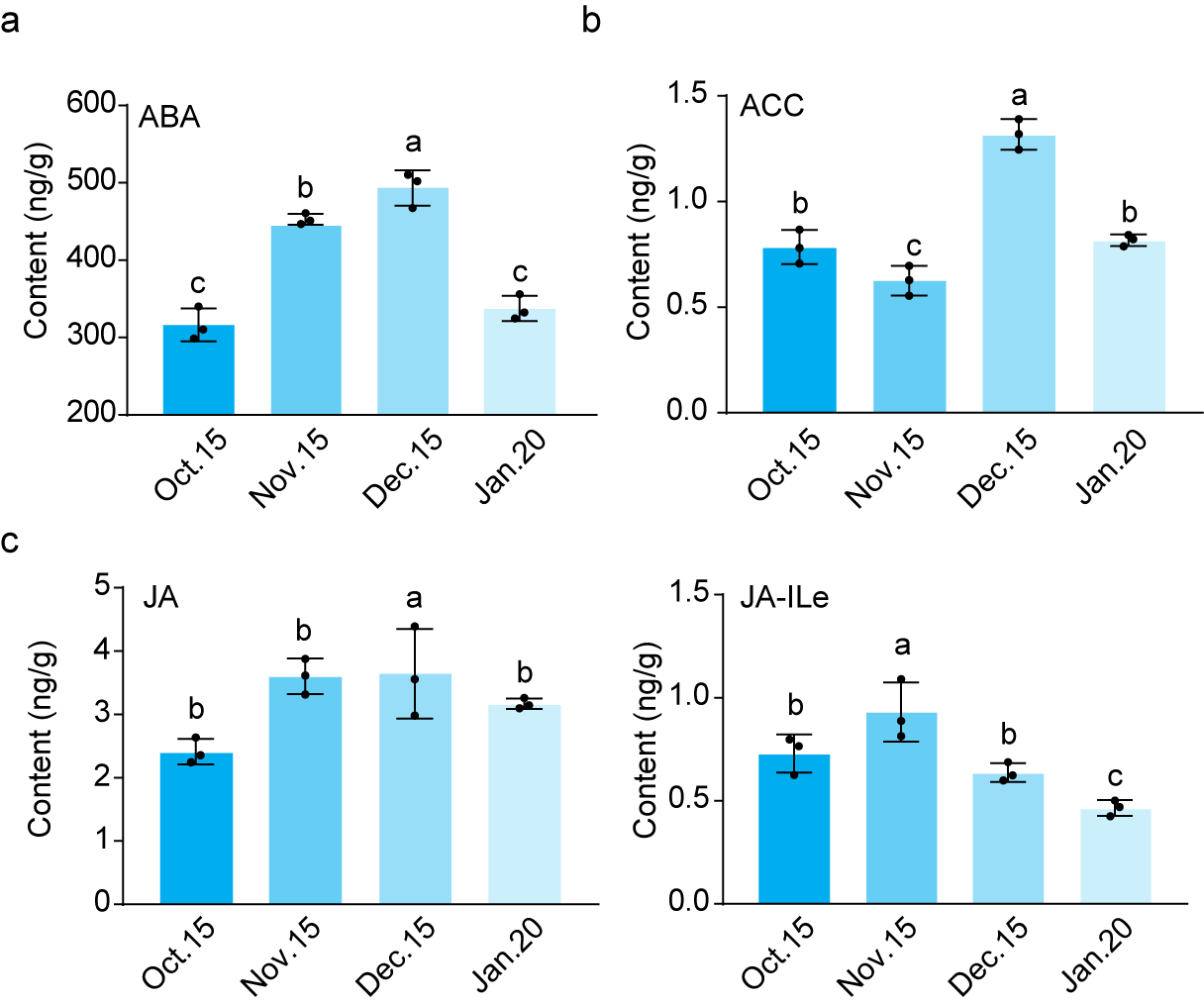


Figure S5 Contents of different kinds of phytohormones in samples under natural condition. Contents of ABA (a), ACC (b), and jasmonates (c). Error bars show the standard deviation of three biological replicates and the dots show the independent values. Different letters indicate significant difference between different samples (one-way ANOVA, *P* < 0.05)


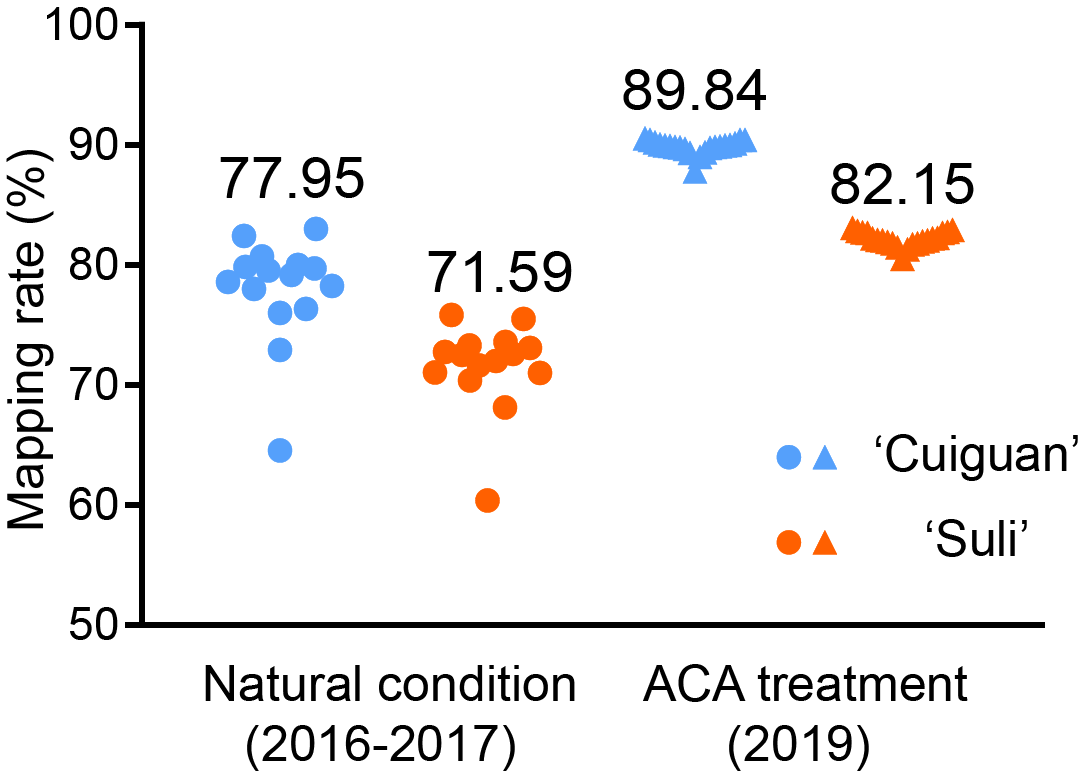


Figure S6 Mapping rates of RNA-seq reads using different pear genome.


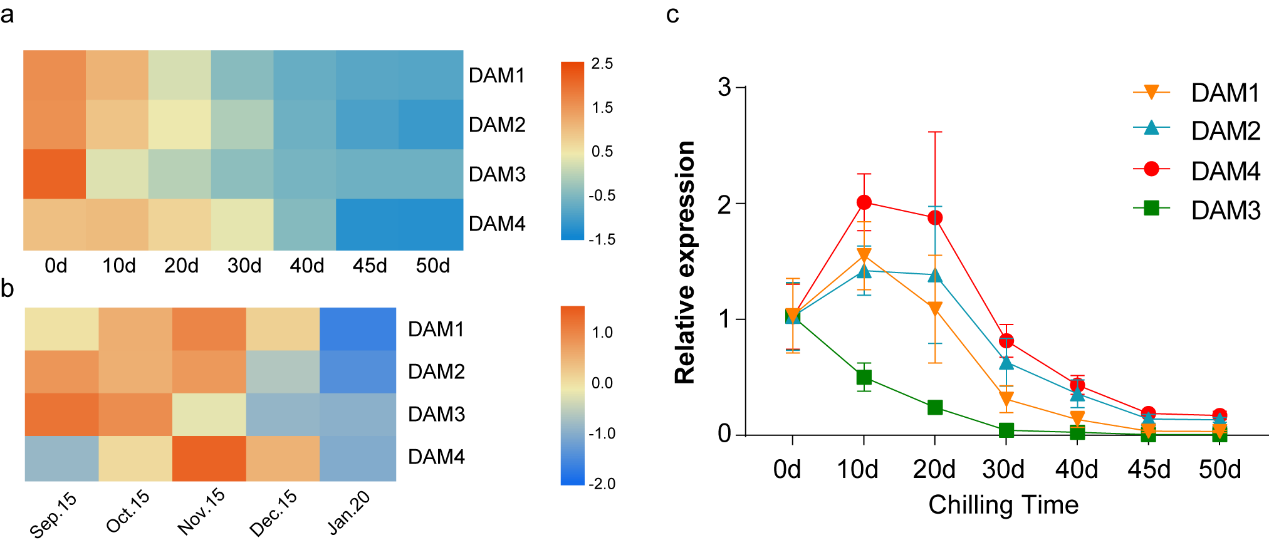


Figure S7 Expression profiles of *DAM* genes. (a) Transcriptome profiles of DAMs in ACA treated samples; (b) Transcriptome profiles of DAMs in natural condition samples; (c) qRT-PCR results showing the relative expression levels of DAMs in ACA treated samples. Error bars show the standard deviation of three biological replicates.


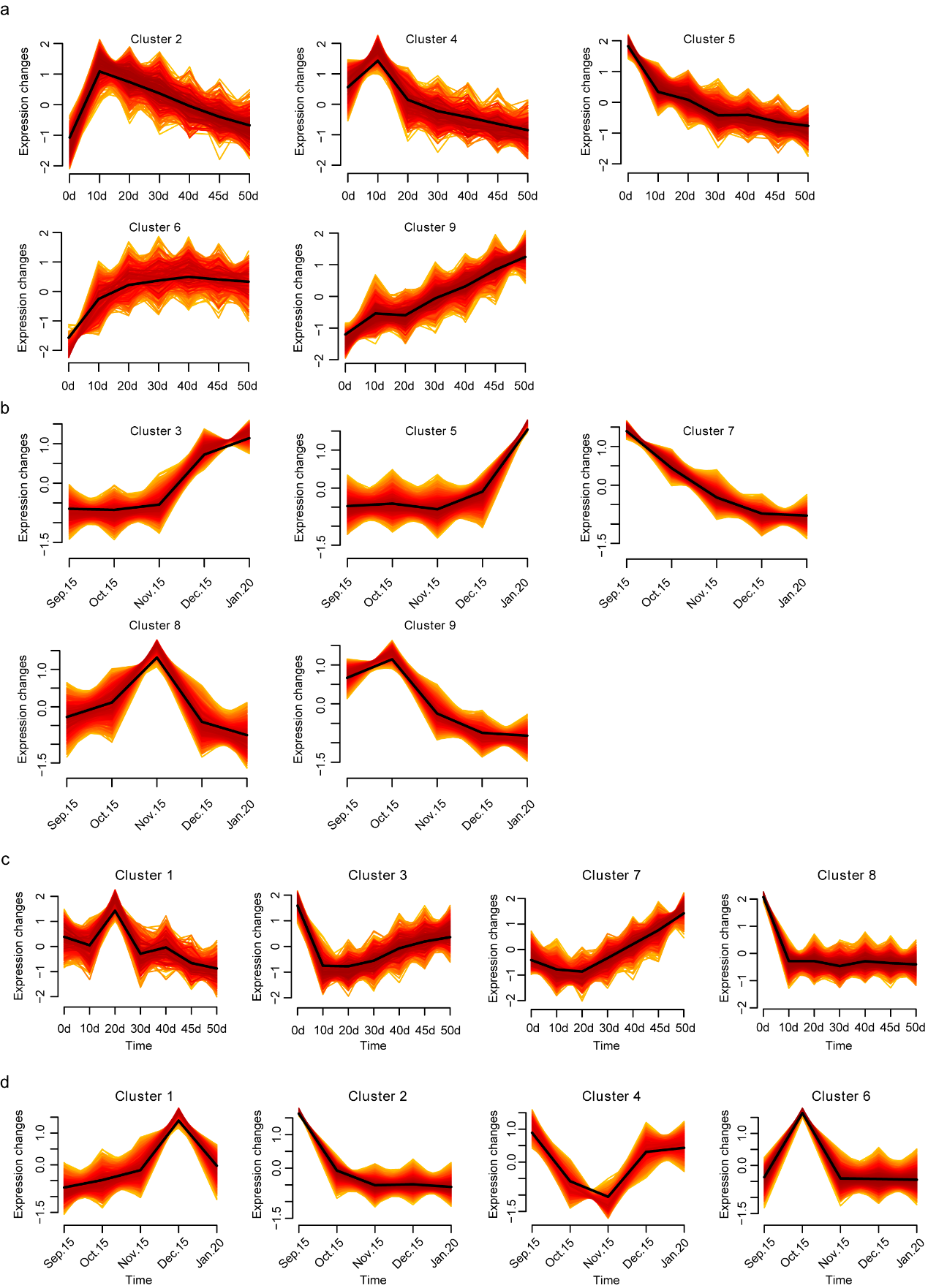


Figure S8 Mfuzz analysis of ACA-treated samples and samples under natural condition. (a) Selected clusters in ACA-treated samples; (b) selected clusters in samples under natural condition; (c) other clusters in ACA-treated samples; (d) other clusters in samples under natural condition.


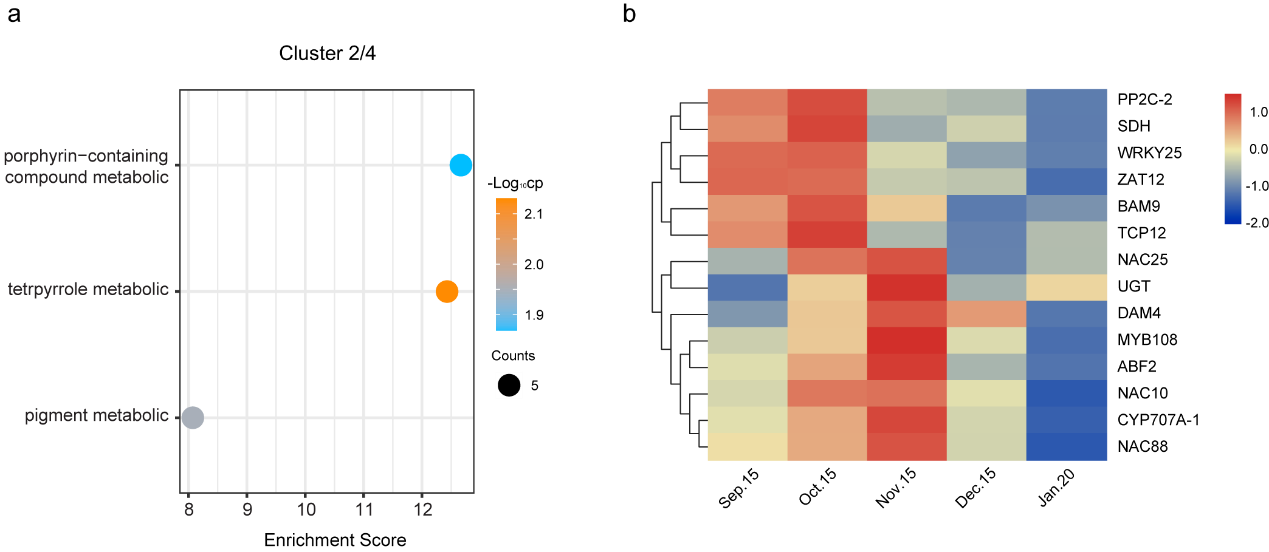


Figure S98 GO analysis of DEGs in ACA-Cluster 2/4 (a) and expression profiles of the DEGs in samples under natural condition samples (b).


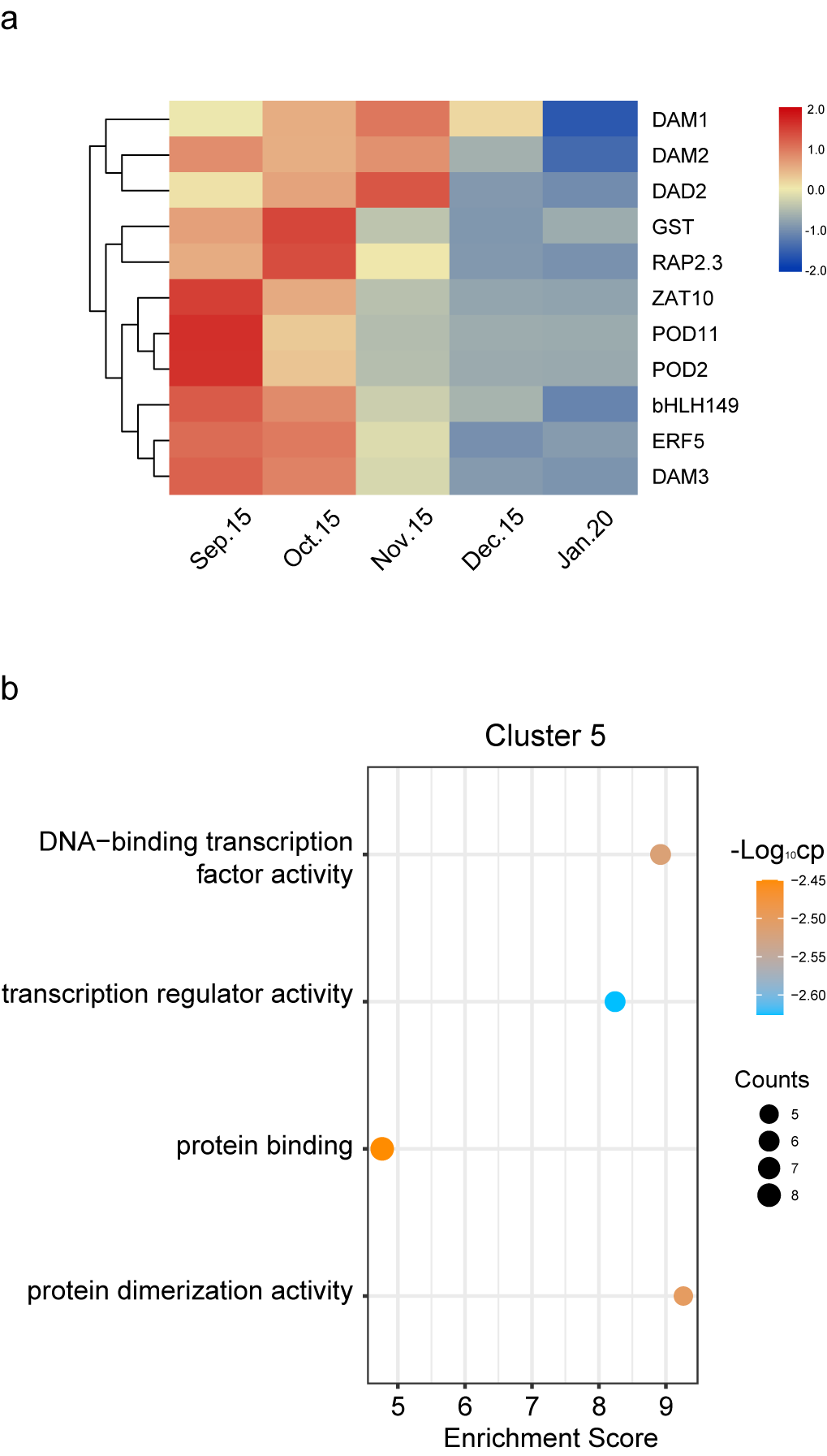


Figure S10 Expression profiles in natural condition samples (a) and GO analysis (b) of DEGs in ACA-Cluster 5.


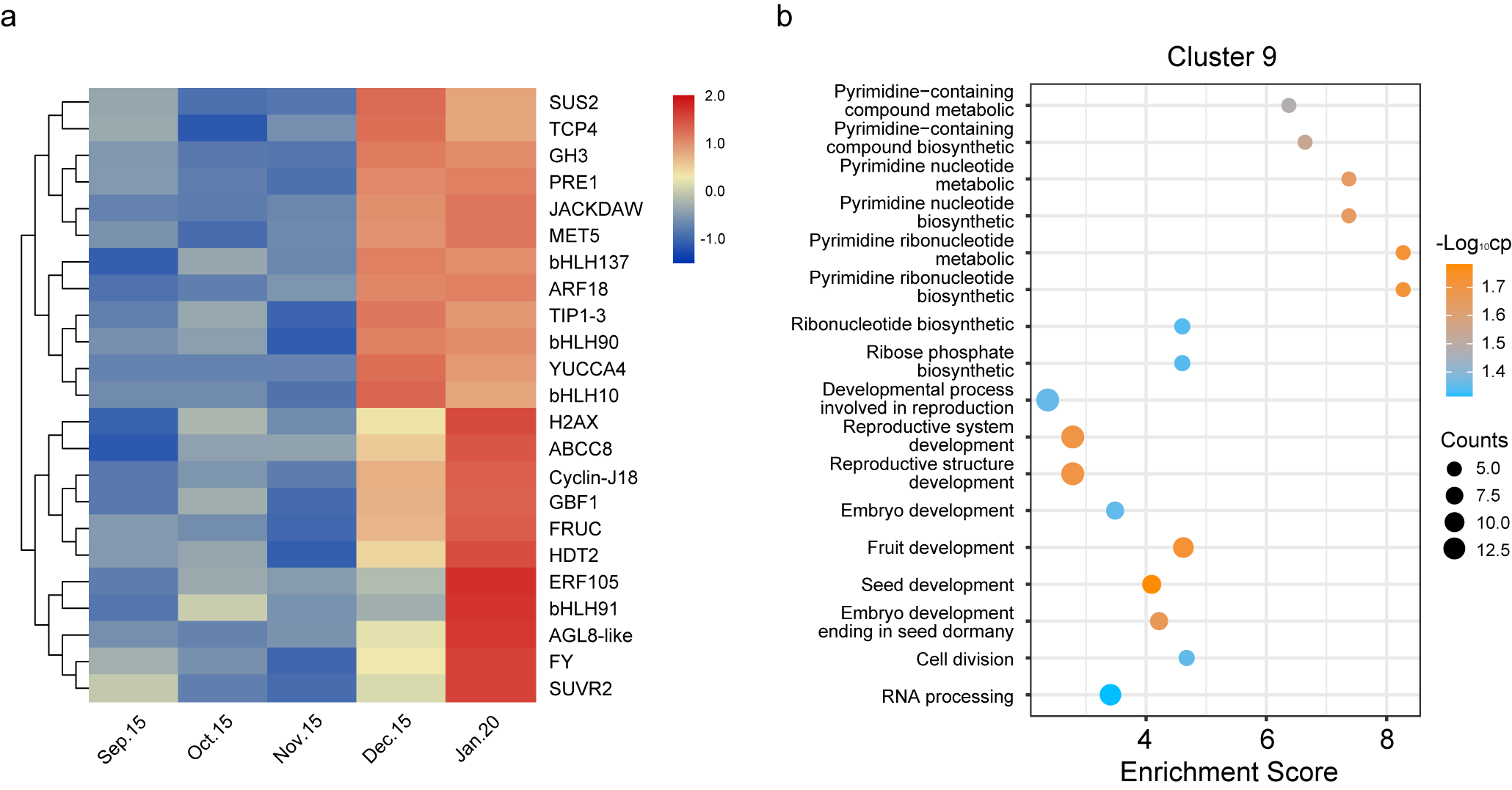


Figure S11 Expression profiles in natural condition samples (a) and GO analysis (b) of DEGs in ACA-Cluster 9.


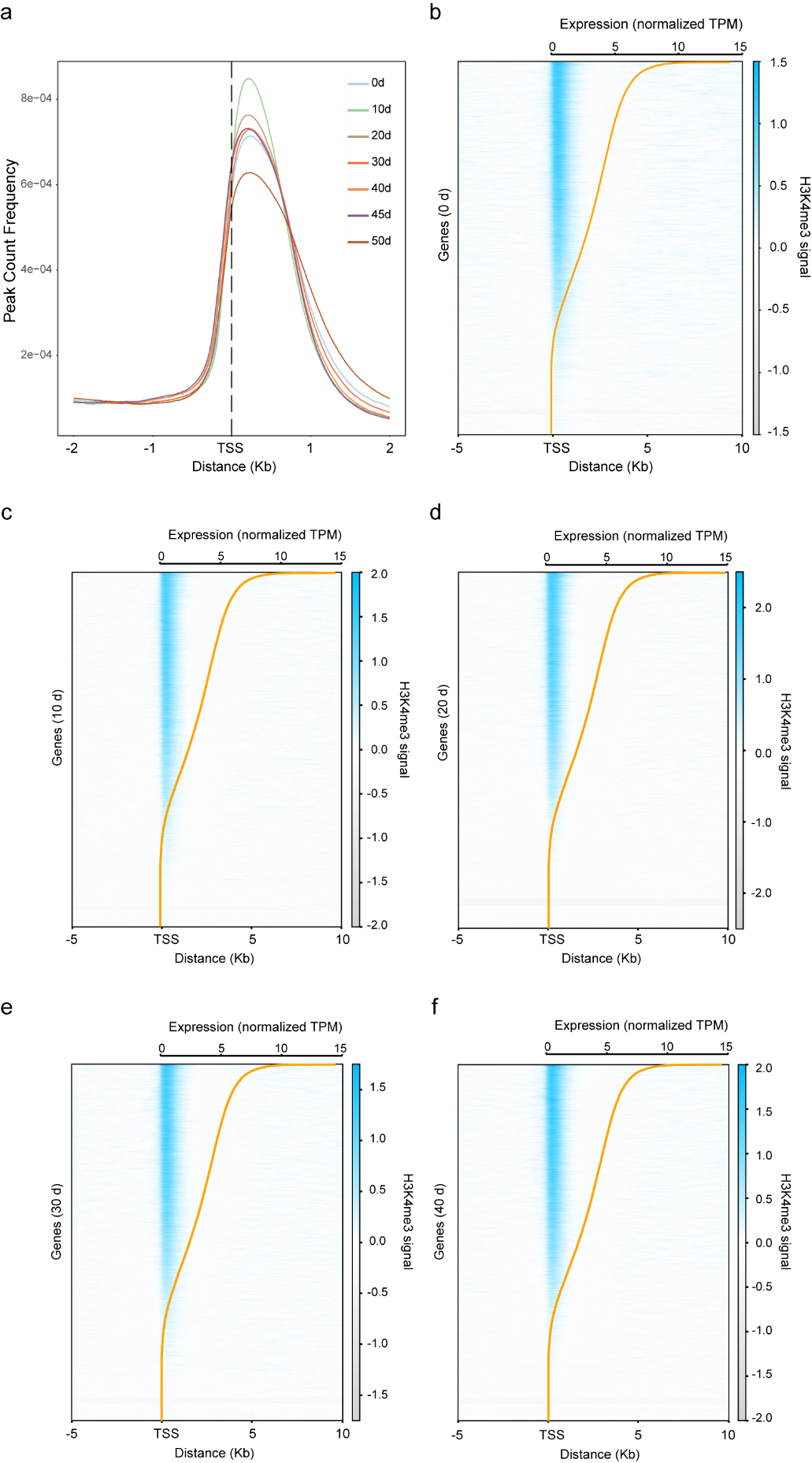


Figure S12 Relationship between the H3K4me3 modification and gene expression. (a) H3K4me3 histone marks are enriched at gene’s TSS in all samples. (b-f) Expression profiles (orange curves) are correlated with H3K4me3 histone modification levels (blue bars) in the whole genome.
