# Supplementary material for: High-quality genome assembly of 'Cuiguan' pear (Pyrus pyrifolia) as a reference genome for identifying regulatory genes and epigenetic modifications responsible for bud dormancy
Source: Hortic Res. 2021 Sep 1;8:197. doi: 10.1038/s41438-021-00632-w (PMC8408243; doi:10.1038/s41438-021-00632-w)
Supplement: Supplementary file 2 — Supplementary experimental procedures [file 41438_2021_632_MOESM2_ESM.docx]

### Methods S1 Phylogenetic analysis

We used Orthofinder (v2.3.3)^1^ to identify orthologous gene clusters, after which phylogenetic relationships were resolved using RAxML (v8.2.11)^2^ based on single-copy gene families aligned with MAFFT (v7.310) ^3^, with 1,000 bootstrap replicates and the accession numbers of genes were available in Table S7. For the phylogenetic analysis of DAMs, we used MEGA X ^4^ to construct a neighbor-joining phylogenetic tree, with 1,000 bootstrap replicates. The MCScanX ^5^ and TBtools ^6^ programs were used to identify collinear gene pairs and calculate Ks values.

### Methods S2 RNA-seq data analysis

Next-generation sequencing reads were mapped to the ‘Cuiguan’ pear genome using HISAT2^7^. Additionally, SAMtools ^8^ was used to convert SAM files into BAM files. The reads covering transcripts were counted with featureCounts ^9^ and then used to identify DEGs with DEseq2 ^10^. Genes with a false discovery rate < 0.1 and log_2_ fold-change ≥ 1 were designated as DEGs. We also calculated the TPM value for each gene. Heatmaps (scaled by row) were prepared and GO enrichment analyses were conducted using TBtools ^6^.

### Methods S3 ChIP-seq data analysis

Following a quality control step, the clean reads were mapped to the ‘Cuiguan’ genome using Bowtie2 (v2.3.5)^11^. The SAMtools program was used to convert SAM files into BAM files. Picard (http://broadinstitute.github.io/picard/) was used to mark and remove PCR-derived duplicates. The log_2_[ratio] bigwig coverage files relative to input were generated using the [bamCompare](https://deeptools.readthedocs.io/en/develop/content/tools/bamCompare.html) function of deepTools ^12^, with a bin size of 10 bp. The bw files were visualized as H3K4me3 signals using Integrative Genomics Viewer (negative values were hidden)^13^ and deepTools. The ChIP-seq peaks were called using MACS2 (v2.1.4) ^14^ and annotated with ChIPseeker ^15^.

**References**

1 Emms, D. M. & Kelly, S. OrthoFinder: solving fundamental biases in whole genome comparisons dramatically improves orthogroup inference accuracy. *Genome Biol.* **16**, 157, doi:10.1186/s13059-015-0721-2 (2015).

2 Stamatakis, A. RAxML version 8: a tool for phylogenetic analysis and post-analysis of large phylogenies. *Bioinformatics* **30**, 1312-1313, doi:10.1093/bioinformatics/btu033 (2014).

3 Katoh, K. & Standley, D. M. MAFFT multiple sequence alignment software version 7: improvements in performance and usability. *Mol. Biol. Evol.* **30**, 772-780, doi:10.1093/molbev/mst010 (2013).

4 Kumar, S., Stecher, G., Li, M., Knyaz, C. & Tamura, K. MEGA X: Molecular Evolutionary Genetics Analysis across Computing Platforms. *Mol. Biol. Evol.* **35**, 1547-1549, doi:10.1093/molbev/msy096 (2018).

5 Wang, Y. *et al.* MCScanX: a toolkit for detection and evolutionary analysis of gene synteny and collinearity. *Nucleic Acids Res.* **40**, e49, doi:10.1093/nar/gkr1293 (2012).

6 Chen, C. *et al.* TBtools - an integrative toolkit developed for interactive analyses of big biological data. *Mol. Plant* **13**, 1194-1202, doi:10.1016/j.molp.2020.06.009 (2020).

7 Kim, D., Langmead, B. & Salzberg, S. L. HISAT: a fast spliced aligner with low memory requirements. *Nat. Methods* **12**, 357-360, doi:10.1038/nmeth.3317 (2015).

8 Li, H. *et al.* The Sequence Alignment/Map format and SAMtools. *Bioinformatics* **25**, 2078-2079, doi:10.1093/bioinformatics/btp352 (2009).

9 Liao, Y., Smyth, G. K. & Shi, W. featureCounts: an efficient general purpose program for assigning sequence reads to genomic features. *Bioinformatics* **30**, 923-930, doi:10.1093/bioinformatics/btt656 (2014).

10 Love, M. I., Huber, W. & Anders, S. Moderated estimation of fold change and dispersion for RNA-seq data with DESeq2. *Genome Biol.* **15**, 550, doi:10.1186/s13059-014-0550-8 (2014).

11 Langmead, B. & Salzberg, S. L. Fast gapped-read alignment with Bowtie 2. *Nat. Methods* **9**, 357-359, doi:10.1038/nmeth.1923 (2012).

12 Ramirez, F. *et al.* deepTools2: a next generation web server for deep-sequencing data analysis. *Nucleic Acids Res.* **44**, W160-165, doi:10.1093/nar/gkw257 (2016).

13 Thorvaldsdottir, H., Robinson, J. T. & Mesirov, J. P. Integrative Genomics Viewer (IGV): high-performance genomics data visualization and exploration. *Brief. Bioinform.* **14**, 178-192, doi:10.1093/bib/bbs017 (2013).

14 Zhang, Y. *et al.* Model-based analysis of ChIP-Seq (MACS). *Genome Biol.* **9**, R137, doi:10.1186/gb-2008-9-9-r137 (2008).

15 Yu, G., Wang, L. G. & He, Q. Y. ChIPseeker: an R/Bioconductor package for ChIP peak annotation, comparison and visualization. *Bioinformatics* **31**, 2382-2383, doi:10.1093/bioinformatics/btv145 (2015).
