# Supplementary material for: High-quality genome assembly of 'Cuiguan' pear (Pyrus pyrifolia) as a reference genome for identifying regulatory genes and epigenetic modifications responsible for bud dormancy
Source: Hortic Res. 2021 Sep 1;8:197. doi: 10.1038/s41438-021-00632-w (PMC8408243; doi:10.1038/s41438-021-00632-w)
Supplement: Supplementary file 3 — Supplementary_tables [file 41438_2021_632_MOESM3_ESM.docx]

**Table S1 Repeat sequence annotation**

| Type | Number | Length | Rate (%) |
| --- | --- | --- | --- |
| ClassI | 562,441 | 249,492,149 | 46.09 |
| ClassI/DIRS | 11,142 | 10,102,497 | 1.87 |
| ClassI/LARD | 129,729 | 27,085,618 | 5 |
| ClassI/LINE | 32,651 | 9,948,619 | 1.84 |
| ClassI/LTR/Copia | 144,594 | 94,612,995 | 17.48 |
| ClassI/LTR/Gypsy | 156,749 | 107,757,401 | 19.91 |
| ClassI/LTR/Unknown | 79,589 | 23,008,854 | 4.25 |
| ClassI/PLE | 1,950 | 964,519 | 0.18 |
| ClassI/SINE | 3,813 | 683,037 | 0.13 |
| ClassI/TRIM | 2,140 | 1,475,054 | 0.27 |
| ClassI/Unknown | 84 | 8,518 | 0 |
| ClassII | 278,359 | 69,719,255 | 12.88 |
| ClassII/Crypton | 12 | 599 | 0 |
| ClassII/Helitron | 40,233 | 10,492,099 | 1.94 |
| ClassII/MITE | 4,925 | 1,163,886 | 0.22 |
| ClassII/Maverick | 2,381 | 455,423 | 0.08 |
| ClassII/TIR | 185,357 | 50,184,682 | 9.27 |
| ClassII/Unknown | 45,451 | 9,674,012 | 1.79 |
| PotentialHostGene | 4,860 | 1,208,996 | 0.22 |
| SSR | 1,325 | 182,734 | 0.03 |
| Unknown | 74,232 | 15,475,166 | 2.86 |
| Total | 921,217 | 314,986,834 | 58.19 |

**Table S2 Gene annotation information**

| Database | Annotated Number | Percentage (%) |
| --- | --- | --- |
| GO Annotation | 21,399 | 50.21% |
| KEGG Annotation | 14,274 | 33.49% |
| KOG Annotation | 23,725 | 55.66% |
| TrEMBL Annotation | 41,720 | 97.88% |
| NR Annotation | 42,247 | 99.12% |
| All Annotated | 42,257 | 99.14% |

| **Table S3 Genes in Cluster 2/4 in ACA-treated samples** | | |
| --- | --- | --- |
| Gene ID | Gene name | Annotation |
| EVM0005767.1 |  | PREDICTED: cystathionine gamma-synthase, chloroplastic-like [Malus domestica] |
| EVM0006086.1 |  | hypothetical protein PRUPE_ppa015491mg, partial [Prunus persica] |
| EVM0007243.1 |  | PREDICTED: protein FANTASTIC FOUR 1-like [Pyrus x bretschneideri] |
| EVM0008666.1 |  | PREDICTED: uncharacterized protein LOC103425055 [Malus domestica] |
| EVM0009480.1 |  | PREDICTED: uncharacterized protein LOC103967087 [Pyrus x bretschneideri] |
| EVM0014056.1 |  | PREDICTED: uncharacterized protein LOC103961788 [Pyrus x bretschneideri] |
| EVM0017455.1 |  | PREDICTED: uncharacterized protein LOC103950045 [Pyrus x bretschneideri] |
| EVM0018385.1 |  | PREDICTED: probable protein phosphatase 2C 8 [Pyrus x bretschneideri] |
| EVM0018602.1 |  | PREDICTED: uncharacterized protein LOC103933654 [Pyrus x bretschneideri] |
| EVM0021265.1 | NAC40 | PREDICTED: NAC transcription factor 25 [Pyrus x bretschneideri] |
| EVM0024589.1 |  | PREDICTED: DEAD-box ATP-dependent RNA helicase 2-like [Pyrus x bretschneideri] |
| EVM0029100.1 |  | PREDICTED: uncharacterized protein LOC103967087 [Pyrus x bretschneideri] |
| EVM0029589.1 |  | PREDICTED: probable protein phosphatase 2C 47 [Pyrus x bretschneideri] |
| EVM0032529.1 | CYP707A-1 | PREDICTED: abscisic acid 8&apos;-hydroxylase 2 [Pyrus x bretschneideri] |
| EVM0032989.1 |  | PREDICTED: uncharacterized protein LOC103421311 [Malus domestica] |
| EVM0033108.1 |  | PREDICTED: protein NIM1-INTERACTING 1 [Pyrus x bretschneideri] |
| EVM0033240.1 |  | PREDICTED: uncharacterized protein LOC103426803 [Malus domestica] |
| EVM0036667.1 |  | PREDICTED: ninja-family protein AFP3-like [Pyrus x bretschneideri] |
| EVM0038107.1 |  | PREDICTED: ninja-family protein AFP3 [Pyrus x bretschneideri] |
| EVM0041987.1 |  | PREDICTED: uncharacterized protein LOC103966573 [Pyrus x bretschneideri] |
| EVM0000361.1 |  | PREDICTED: uncharacterized protein LOC103426647 isoform X1 [Malus domestica] |
| EVM0001958.1 |  | PREDICTED: uncharacterized protein LOC103944978 [Pyrus x bretschneideri] |
| EVM0002377.1 |  | PREDICTED: late embryogenesis abundant protein, group 3-like isoform X2 [Pyrus x bretschneideri] |
| EVM0003919.1 |  | PREDICTED: oleosin 1-like [Pyrus x bretschneideri] |
| EVM0004408.1 |  | PREDICTED: thylakoid lumenal 17.4 kDa protein, chloroplastic-like [Malus domestica] |
| EVM0005540.1 |  | PREDICTED: protein phosphatase 2C 37-like [Pyrus x bretschneideri] |
| EVM0005957.1 |  | PREDICTED: bicaudal D-related protein 2-like [Malus domestica] |
| EVM0007312.1 |  | PREDICTED: uncharacterized protein LOC103950685 [Pyrus x bretschneideri] |
| EVM0008089.1 |  | PREDICTED: uncharacterized protein LOC103927052 [Pyrus x bretschneideri] |
| EVM0008298.1 | PP2C-2 | PREDICTED: protein phosphatase 2C 77-like isoform X1 [Pyrus x bretschneideri] |
| EVM0008391.1 |  | PREDICTED: F-box/kelch-repeat protein At2g44130-like [Pyrus x bretschneideri] |
| EVM0010824.1 |  | PREDICTED: putative glycine-rich cell wall structural protein 1 [Malus domestica] |
| EVM0012159.1 | NAC88 | PREDICTED: NAC domain-containing protein 72 [Pyrus x bretschneideri] |
| EVM0012380.1 |  | PREDICTED: suppressor protein SRP40-like isoform X1 [Pyrus x bretschneideri] |
| EVM0013639.1 |  | PREDICTED: homocysteine S-methyltransferase 3 [Pyrus x bretschneideri] |
| EVM0015642.1 | MYB108 | PREDICTED: transcription factor MYB108-like [Pyrus x bretschneideri] |
| EVM0015929.1 |  | PREDICTED: uncharacterized protein LOC103966037 [Pyrus x bretschneideri] |
| EVM0016984.1 |  | PREDICTED: geranylgeranyl diphosphate reductase, chloroplastic-like [Malus domestica] |
| EVM0017091.1 |  | PREDICTED: tonoplast dicarboxylate transporter-like [Pyrus x bretschneideri] |
| EVM0017855.1 |  | PREDICTED: chlorophyll a-b binding protein CP29.1, chloroplastic [Pyrus x bretschneideri] |
| EVM0018663.1 |  | PREDICTED: translocator protein homolog [Malus domestica] |
| EVM0019008.1 |  | hypothetical protein PRUPE_ppb009453mg [Prunus persica] |
| EVM0019031.1 |  | PREDICTED: LOW QUALITY PROTEIN: minor allergen Alt a 7-like [Malus domestica] |
| EVM0019565.1 |  | PREDICTED: probable glycosyltransferase At5g03795 [Prunus mume] |
| EVM0022070.1 |  | PREDICTED: thylakoid lumenal 17.4 kDa protein, chloroplastic-like [Malus domestica] |
| EVM0022634.1 |  | PREDICTED: glutamate receptor 1.2-like [Malus domestica] |
| EVM0023343.1 |  | PREDICTED: sugar carrier protein C [Pyrus x bretschneideri] |
| EVM0025606.1 |  | hypothetical protein JCGZ_24809 [Jatropha curcas] |
| EVM0025682.1 |  | PREDICTED: probable alpha,alpha-trehalose-phosphate synthase [UDP-forming] 10 [Malus domestica] |
| EVM0026227.1 | MAPKKK | PREDICTED: mitogen-activated protein kinase kinase kinase A-like [Pyrus x bretschneideri] |
| EVM0026501.1 | UGT | PREDICTED: anthocyanidin 3-O-glucosyltransferase 2-like [Pyrus x bretschneideri] |
| EVM0027818.2 |  | PREDICTED: beta-carotene hydroxylase 2, chloroplastic-like [Pyrus x bretschneideri] |
| EVM0028746.1 |  | PREDICTED: transmembrane ascorbate ferrireductase 1-like [Pyrus x bretschneideri] |
| EVM0028804.1 |  | PREDICTED: uncharacterized protein LOC103935988 [Pyrus x bretschneideri] |
| EVM0029523.1 |  | PREDICTED: dynein light chain-like [Pyrus x bretschneideri] |
| EVM0029736.1 | WRKY75 | PREDICTED: probable WRKY transcription factor 75 [Pyrus x bretschneideri] |
| EVM0030821.1 |  | PREDICTED: 4-hydroxyphenylpyruvate dioxygenase-like [Pyrus x bretschneideri] |
| EVM0032747.1 | ZAT12 | PREDICTED: zinc finger protein ZAT12-like [Pyrus x bretschneideri] |
| EVM0033154.1 | SDH | PREDICTED: sorbitol dehydrogenase-like [Pyrus x bretschneideri] |
| EVM0033910.1 |  | PREDICTED: heme oxygenase 1, chloroplastic-like [Pyrus x bretschneideri] |
| EVM0034269.1 |  | PREDICTED: phytoene synthase 2, chloroplastic-like [Pyrus x bretschneideri] |
| EVM0034889.1 |  | PREDICTED: histone H3.v1 [Pyrus x bretschneideri] |
| EVM0036139.1 |  | PREDICTED: E3 ubiquitin-protein ligase ZNRF3-like isoform X1 [Prunus mume] |
| EVM0037968.1 |  | PREDICTED: uncharacterized protein LOC103452412 [Malus domestica] |
| EVM0038218.1 | BAM9 | PREDICTED: inactive beta-amylase 9-like [Pyrus x bretschneideri] |
| EVM0038431.1 |  | PREDICTED: RING-H2 finger protein ATL38-like [Pyrus x bretschneideri] |
| EVM0039176.1 | ABF2 | PREDICTED: ABSCISIC ACID-INSENSITIVE 5-like protein 5 isoform X1 [Pyrus x bretschneideri] |
| EVM0039778.1 |  | PREDICTED: probable disease resistance protein At5g66900 [Pyrus x bretschneideri] |
| EVM0004250.1 | DAM4 | dormancy-associated MADS-box transcription factor [Pyrus pyrifolia var. culta] |
| EVM0039955.1 |  | PREDICTED: dirigent protein 25 [Malus domestica] |
| EVM0041096.1 | TCP12 | PREDICTED: transcription factor TCP12-like [Pyrus x bretschneideri] |
| EVM0042383.1 | NAC10 | PREDICTED: LOW QUALITY PROTEIN: NAC domain-containing protein 72, partial [Pyrus x bretschneideri] |
| EVM0042521.1 |  | PREDICTED: uncharacterized protein LOC103947353 [Pyrus x bretschneideri] |

| **Table S4 Genes in Cluster 5 in ACA-treated samples** | | |
| --- | --- | --- |
| Gene ID | Gene name | Annotation |
| EVM0000124.1 |  | PREDICTED: light-regulated protein-like [Pyrus x bretschneideri] |
| EVM0001467.1 |  | PREDICTED: uncharacterized protein LOC103944736 [Pyrus x bretschneideri] |
| EVM0001492.1 |  | PREDICTED: non-specific lipid-transfer protein-like protein At2g13820 isoform X2 [Pyrus x bretschneideri] |
| EVM0002291.1 | ZAT10 | PREDICTED: zinc finger protein ZAT10 [Pyrus x bretschneideri] |
| EVM0004339.1 |  | PREDICTED: uncharacterized protein LOC103448526 [Malus domestica] |
| EVM0005068.1 |  | PREDICTED: dynein light chain 1, cytoplasmic-like [Pyrus x bretschneideri] |
| EVM0005100.1 | GST | PREDICTED: probable glutathione S-transferase isoform X2 [Pyrus x bretschneideri] |
| EVM0006495.1 |  | PREDICTED: photosystem II 10 kDa polypeptide, chloroplastic-like [Pyrus x bretschneideri] |
| EVM0006581.1 |  | PREDICTED: F-box protein At1g61340-like [Pyrus x bretschneideri] |
| EVM0007934.1 |  | PREDICTED: probable protein phosphatase 2C 49 [Pyrus x bretschneideri] |
| EVM0009473.1 | DAM2 | PREDICTED: MADS-box protein AGL24-like [Pyrus x bretschneideri] |
| EVM0009605.1 |  | PREDICTED: proline-rich receptor-like protein kinase PERK2 [Malus domestica] |
| EVM0010506.1 | bHLH149 | PREDICTED: transcription factor bHLH149-like [Pyrus x bretschneideri] |
| EVM0010779.1 |  | PREDICTED: uncharacterized protein LOC103452793 [Malus domestica] |
| EVM0011129.1 |  | PREDICTED: high-light-induced protein, chloroplastic [Malus domestica] |
| EVM0013227.1 |  | BHLH domain class transcription factor [Malus domestica] |
| EVM0013767.1 |  | PREDICTED: proline-rich receptor-like protein kinase PERK2 [Malus domestica] |
| EVM0013779.1 |  | PREDICTED: asparagine synthetase [glutamine-hydrolyzing] [Pyrus x bretschneideri] |
| EVM0013830.1 |  | PREDICTED: uncharacterized protein LOC105119217 [Populus euphratica] |
| EVM0014249.1 |  | PREDICTED: uncharacterized protein LOC103957865 [Pyrus x bretschneideri] |
| EVM0015109.1 | DAM1 | PREDICTED: MADS-box protein AGL24-like [Pyrus x bretschneideri] |
| EVM0015656.1 |  | PREDICTED: laccase-12-like [Malus domestica] |
| EVM0015957.1 |  | PREDICTED: uncharacterized protein LOC103940076 [Pyrus x bretschneideri] |
| EVM0016233.1 |  | PREDICTED: CASP-like protein VIT_05s0020g01830 [Pyrus x bretschneideri] |
| EVM0016665.1 | DAD2 | PREDICTED: probable strigolactone esterase DAD2 [Pyrus x bretschneideri] |
| EVM0019356.1 |  | PREDICTED: photosystem I reaction center subunit VI-2, chloroplastic [Pyrus x bretschneideri] |
| EVM0020400.1 |  | PREDICTED: uncharacterized protein LOC103948529 [Pyrus x bretschneideri] |
| EVM0020641.1 |  | PREDICTED: photosystem I subunit O-like [Pyrus x bretschneideri] |
| EVM0021300.1 |  | PREDICTED: GDSL esterase/lipase At1g74460-like [Pyrus x bretschneideri] |
| EVM0021496.1 |  | PREDICTED: uncharacterized protein LOC103930917 [Pyrus x bretschneideri] |
| EVM0021497.1 |  | PREDICTED: uncharacterized protein LOC103423377 [Malus domestica] |
| EVM0021511.1 |  | PREDICTED: blue copper protein [Malus domestica] |
| EVM0022924.1 |  | PREDICTED: non-specific lipid-transfer protein-like protein At2g13820 isoform X2 [Pyrus x bretschneideri] |
| EVM0023048.1 |  | PREDICTED: RING-H2 finger protein ATL2-like [Pyrus x bretschneideri] |
| EVM0023062.1 | ERF5 | PREDICTED: ethylene-responsive transcription factor 5-like [Pyrus x bretschneideri] |
| EVM0024106.1 |  | PREDICTED: nematode resistance protein-like HSPRO2 [Pyrus x bretschneideri] |
| EVM0025109.1 |  | PREDICTED: cytochrome P450 86A1-like [Malus domestica] |
| EVM0025833.1 |  | PREDICTED: probable calcium-binding protein CML36 [Malus domestica] |
| EVM0028042.1 | POD11 | PREDICTED: peroxidase 11-like [Pyrus x bretschneideri] |
| EVM0029327.1 |  | PREDICTED: dirigent protein 22-like [Pyrus x bretschneideri] |
| EVM0030596.1 |  | PREDICTED: chlorophyll a-b binding protein 8, chloroplastic [Malus domestica] |
| EVM0033157.1 | RAP2.3 | PREDICTED: ethylene-responsive transcription factor RAP2-3 isoform X1 [Pyrus x bretschneideri] |
| EVM0034366.1 |  | PREDICTED: glyceraldehyde-3-phosphate dehydrogenase A, chloroplastic isoform X1 [Pyrus x bretschneideri] |
| EVM0035466.1 |  | PREDICTED: CASP-like protein POPTRDRAFT_823125 [Pyrus x bretschneideri] |
| EVM0035713.1 |  | PREDICTED: uncharacterized protein LOC103933717 [Pyrus x bretschneideri] |
| EVM0036752.1 |  | PREDICTED: calcium-binding protein CML42-like [Pyrus x bretschneideri] |
| EVM0037239.1 |  | PREDICTED: chlorophyll a-b binding protein P4, chloroplastic [Pyrus x bretschneideri] |
| EVM0038346.1 |  | PREDICTED: photosystem II repair protein PSB27-H1, chloroplastic-like [Malus domestica] |
| EVM0038385.1 |  | PREDICTED: uncharacterized protein DDB_G0271670-like [Pyrus x bretschneideri] |
| EVM0038867.1 |  | PREDICTED: cysteine-rich and transmembrane domain-containing protein B-like isoform X1 [Pyrus x bretschneideri] |
| EVM0038901.1 |  | PREDICTED: uncharacterized protein LOC103929813 [Pyrus x bretschneideri] |
| EVM0039886.1 |  | PREDICTED: LOW QUALITY PROTEIN: EID1-like F-box protein 3 [Malus domestica] |
| EVM0039962.1 |  | PREDICTED: uncharacterized protein LOC103424445 [Malus domestica] |
| EVM0040090.1 |  | PREDICTED: fasciclin-like arabinogalactan protein 11 [Pyrus x bretschneideri] |
| EVM0040391.1 |  | PREDICTED: MADS-box protein AGL24-like [Pyrus x bretschneideri] |
| EVM0040583.1 |  | PREDICTED: 2-alkenal reductase (NADP(+)-dependent)-like [Pyrus x bretschneideri] |
| EVM0040868.1 | POD2 | PREDICTED: peroxidase-like protein 2 [Malus domestica] |
| EVM0041302.1 | DAM3 | PREDICTED: MADS-box protein AGL24-like [Pyrus x bretschneideri] |

| **Table S5 Genes in Cluster 9 in ACA-treated samples** | | |
| --- | --- | --- |
| Gene ID | Gene Name | Annotation |
| EVM0000171.2 |  | PREDICTED: protein RRP45A-like isoform X1 [Malus domestica] |
| EVM0000605.1 | YUCCA4 | PREDICTED: probable indole-3-pyruvate monooxygenase YUCCA4 [Pyrus x bretschneideri] |
| EVM0000639.1 |  | PREDICTED: S-adenosylmethionine decarboxylase proenzyme [Pyrus x bretschneideri] |
| EVM0000777.1 | CYCLIN-J18 | PREDICTED: cyclin-J18 [Malus domestica] |
| EVM0001615.1 |  | PREDICTED: guanine nucleotide-binding protein subunit gamma 1-like [Pyrus x bretschneideri] |
| EVM0001833.1 |  | PREDICTED: bifunctional epoxide hydrolase 2-like [Pyrus x bretschneideri] |
| EVM0001936.1 |  | PREDICTED: tRNA dimethylallyltransferase 9-like [Malus domestica] |
| EVM0001948.1 |  | PREDICTED: queuine tRNA-ribosyltransferase-like [Pyrus x bretschneideri] |
| EVM0002112.1 |  | PREDICTED: B3 domain-containing protein REM8-like [Malus domestica] |
| EVM0002210.1 |  | PREDICTED: protein NLRC3-like [Malus domestica] |
| EVM0002227.1 |  | PREDICTED: uncharacterized protein LOC103955197 [Pyrus x bretschneideri] |
| EVM0002495.1 |  | PREDICTED: uncharacterized protein LOC103940828 [Pyrus x bretschneideri] |
| EVM0002638.1 |  | PREDICTED: DEAD-box ATP-dependent RNA helicase 18-like [Pyrus x bretschneideri] |
| EVM0003073.1 |  | PREDICTED: LOW QUALITY PROTEIN: uncharacterized protein LOC103435116 [Malus domestica] |
| EVM0003463.1 |  | PREDICTED: uncharacterized protein LOC103951009 [Pyrus x bretschneideri] |
| EVM0003742.1 |  | PREDICTED: uncharacterized protein LOC103951073 [Pyrus x bretschneideri] |
| EVM0003781.1 |  | PREDICTED: uncharacterized protein LOC103955887 isoform X1 [Pyrus x bretschneideri] |
| EVM0004017.1 |  | PREDICTED: chaperonin 60 subunit alpha 2, chloroplastic-like [Malus domestica] |
| EVM0004308.1 |  | PREDICTED: DEAD-box ATP-dependent RNA helicase 51-like [Malus domestica] |
| EVM0004472.1 |  | PREDICTED: geraniol 8-hydroxylase-like [Pyrus x bretschneideri] |
| EVM0004514.1 |  | PREDICTED: nardilysin-like isoform X1 [Pyrus x bretschneideri] |
| EVM0005137.1 |  | PREDICTED: serine/threonine-protein kinase HT1-like [Malus domestica] |
| EVM0005272.1 |  | PREDICTED: K(+) efflux antiporter 3, chloroplastic-like isoform X1 [Pyrus x bretschneideri] |
| EVM0005273.1 |  | PREDICTED: aquaporin TIP1-3-like [Pyrus x bretschneideri] |
| EVM0005351.1 |  | PREDICTED: ribonuclease II, chloroplastic/mitochondrial-like [Pyrus x bretschneideri] |
| EVM0005421.1 |  | PREDICTED: uncharacterized protein LOC103951743 [Pyrus x bretschneideri] |
| EVM0005576.1 |  | PREDICTED: chaperone protein ClpB4, mitochondrial [Malus domestica] |
| EVM0005876.1 |  | PREDICTED: 21 kDa protein-like [Pyrus x bretschneideri] |
| EVM0006211.1 | FY | PREDICTED: flowering time control protein FY-like [Pyrus x bretschneideri] |
| EVM0006814.1 |  | PREDICTED: uncharacterized protein LOC103935727 [Pyrus x bretschneideri] |
| EVM0006835.1 |  | PREDICTED: LOW QUALITY PROTEIN: putative CCA tRNA nucleotidyltransferase 2 [Pyrus x bretschneideri] |
| EVM0006884.1 |  | PREDICTED: uridine-cytidine kinase C-like [Pyrus x bretschneideri] |
| EVM0006954.1 |  | PREDICTED: heat shock 70 kDa protein 8-like [Malus domestica] |
| EVM0007090.1 |  | PREDICTED: uncharacterized protein LOC103966128 [Pyrus x bretschneideri] |
| EVM0007157.1 |  | PREDICTED: high mobility group B protein 7-like isoform X1 [Pyrus x bretschneideri] |
| EVM0007236.1 |  | PREDICTED: uncharacterized protein LOC103946543 [Pyrus x bretschneideri] |
| EVM0007287.1 |  | PREDICTED: uncharacterized protein LOC103947321 [Pyrus x bretschneideri] |
| EVM0007750.1 |  | PREDICTED: oxysterol-binding protein-related protein 4B-like [Malus domestica] |
| EVM0007780.1 |  | PREDICTED: DNA repair protein RAD50 [Pyrus x bretschneideri] |
| EVM0008499.1 |  | PREDICTED: hsp70 nucleotide exchange factor fes1-like [Pyrus x bretschneideri] |
| EVM0008840.1 |  | PREDICTED: small RNA degrading nuclease 5-like [Pyrus x bretschneideri] |
| EVM0009585.1 |  | PREDICTED: cation/H(+) antiporter 2-like [Malus domestica] |
| EVM0009803.1 |  | PREDICTED: uncharacterized protein LOC103400552 [Malus domestica] |
| EVM0009880.1 |  | PREDICTED: ABC transporter G family member 28-like isoform X1 [Pyrus x bretschneideri] |
| EVM0010163.1 |  | PREDICTED: uncharacterized protein LOC103958625 [Pyrus x bretschneideri] |
| EVM0010261.1 |  | ATP synthase F0 subunit 6 (mitochondrion) [Malus domestica] |
| EVM0010413.1 |  | PREDICTED: receptor-like protein 2 isoform X1 [Malus domestica] |
| EVM0010507.1 |  | PREDICTED: MATH domain and coiled-coil domain-containing protein At3g58210-like [Pyrus x bretschneideri] |
| EVM0010538.1 |  | PREDICTED: T-complex protein 1 subunit theta-like [Pyrus x bretschneideri] |
| EVM0010633.1 |  | PREDICTED: polygalacturonase-like [Pyrus x bretschneideri] |
| EVM0011239.1 |  | PREDICTED: beta-xylosidase/alpha-L-arabinofuranosidase 1-like [Pyrus x bretschneideri] |
| EVM0011641.2 |  | PREDICTED: uncharacterized protein LOC103949378 [Pyrus x bretschneideri] |
| EVM0011923.1 |  | #N/A |
| EVM0012180.1 |  | PREDICTED: uncharacterized protein LOC103932724 isoform X1 [Pyrus x bretschneideri] |
| EVM0012283.1 |  | PREDICTED: probable ATP-dependent DNA helicase HFM1 [Pyrus x bretschneideri] |
| EVM0012621.1 |  | PREDICTED: uncharacterized protein LOC103423952 isoform X2 [Malus domestica] |
| EVM0012695.1 |  | PREDICTED: LON peptidase N-terminal domain and RING finger protein 1-like [Pyrus x bretschneideri] |
| EVM0013106.1 | GH3 | PREDICTED: glucan endo-1,3-beta-glucosidase 3-like [Malus domestica] |
| EVM0013550.1 |  | PREDICTED: probable polyamine oxidase 5 [Pyrus x bretschneideri] |
| EVM0013574.1 |  | PREDICTED: probable LRR receptor-like serine/threonine-protein kinase At3g47570 [Malus domestica] |
| EVM0013636.1 |  | PREDICTED: uncharacterized protein LOC103945776 [Pyrus x bretschneideri] |
| EVM0013678.1 |  | PREDICTED: codeine O-demethylase [Pyrus x bretschneideri] |
| EVM0013755.1 |  | PREDICTED: RNA polymerase II subunit A C-terminal domain phosphatase SSU72-like [Pyrus x bretschneideri] |
| EVM0014090.1 |  | PREDICTED: glutamate dehydrogenase 1-like isoform X2 [Pyrus x bretschneideri] |
| EVM0014190.1 |  | PREDICTED: uncharacterized protein LOC103408204 [Malus domestica] |
| EVM0014264.1 |  | PREDICTED: uncharacterized protein LOC103937814 [Pyrus x bretschneideri] |
| EVM0014533.1 |  | PREDICTED: monothiol glutaredoxin-S6-like [Malus domestica] |
| EVM0014709.1 |  | PREDICTED: tetratricopeptide repeat protein 27 homolog [Pyrus x bretschneideri] |
| EVM0014822.1 |  | cytochrome c oxidase subunit 1, partial (mitochondrion) [Fragaria iinumae] |
| EVM0015272.1 |  | PREDICTED: WD repeat-containing protein 55-like [Malus domestica] |
| EVM0015695.1 |  | PREDICTED: uncharacterized protein LOC103964549 [Pyrus x bretschneideri] |
| EVM0015703.1 |  | PREDICTED: ecotropic viral integration site 5 protein homolog [Malus domestica] |
| EVM0015803.1 |  | PREDICTED: uncharacterized protein LOC103957596 [Pyrus x bretschneideri] |
| EVM0016141.1 |  | PREDICTED: probable boron transporter 6 isoform X2 [Pyrus x bretschneideri] |
| EVM0016202.1 |  | PREDICTED: GDSL esterase/lipase At5g08460 [Pyrus x bretschneideri] |
| EVM0016523.1 |  | PREDICTED: LOB domain-containing protein 27-like [Pyrus x bretschneideri] |
| EVM0016528.1 | PRE1 | PREDICTED: transcription factor PRE1 [Malus domestica] |
| EVM0016925.1 |  | PREDICTED: uncharacterized protein LOC103403539 isoform X2 [Malus domestica] |
| EVM0017508.1 |  | PREDICTED: uncharacterized protein LOC103410201 [Malus domestica] |
| EVM0017804.1 |  | PREDICTED: U3 small nucleolar RNA-associated protein 25 [Malus domestica] |
| EVM0018035.1 |  | PREDICTED: organ-specific protein P4-like [Pyrus x bretschneideri] |
| EVM0018391.1 | ERF015 | PREDICTED: ethylene-responsive transcription factor ERF015 [Pyrus x bretschneideri] |
| EVM0018833.1 |  | PREDICTED: uncharacterized protein LOC103400906 [Malus domestica] |
| EVM0019036.1 |  | PREDICTED: uncharacterized protein LOC103958761 [Pyrus x bretschneideri] |
| EVM0019164.1 |  | PREDICTED: uncharacterized protein LOC103949811 [Pyrus x bretschneideri] |
| EVM0019435.1 |  | PREDICTED: probable glycerol-3-phosphate acyltransferase 8 [Pyrus x bretschneideri] |
| EVM0019938.1 |  | PREDICTED: NHL repeat-containing protein 2 [Pyrus x bretschneideri] |
| EVM0019946.1 |  | PREDICTED: uncharacterized ATP-dependent helicase C29A10.10c-like [Prunus mume] |
| EVM0019976.1 |  | #N/A |
| EVM0020034.1 |  | PREDICTED: MATE efflux family protein 2, chloroplastic isoform X1 [Pyrus x bretschneideri] |
| EVM0020537.1 |  | PREDICTED: B3 domain-containing protein REM9-like [Pyrus x bretschneideri] |
| EVM0020726.1 |  | PREDICTED: uncharacterized protein LOC103959537 isoform X1 [Pyrus x bretschneideri] |
| EVM0021102.1 |  | PREDICTED: armadillo repeat-containing kinesin-like protein 1 [Pyrus x bretschneideri] |
| EVM0021155.1 |  | PREDICTED: CST complex subunit CTC1 [Malus domestica] |
| EVM0021167.1 |  | PREDICTED: probable ATP-dependent DNA helicase HFM1 [Pyrus x bretschneideri] |
| EVM0021314.1 |  | PREDICTED: uncharacterized protein LOC103945561 [Pyrus x bretschneideri] |
| EVM0021442.1 |  | PREDICTED: zinc finger protein JACKDAW-like [Pyrus x bretschneideri] |
| EVM0021570.1 |  | PREDICTED: cytochrome P450 704C1-like [Pyrus x bretschneideri] |
| EVM0021758.1 | AGL8 | PREDICTED: truncated transcription factor CAULIFLOWER A-like [Pyrus x bretschneideri] |
| EVM0021800.1 |  | PREDICTED: neurochondrin [Pyrus x bretschneideri] |
| EVM0022360.1 | bHLH10-like | PREDICTED: transcription factor bHLH10-like [Pyrus x bretschneideri] |
| EVM0022465.1 |  | PREDICTED: inactive tetrahydrocannabinolic acid synthase-like [Pyrus x bretschneideri] |
| EVM0022539.1 | H2AX | PREDICTED: histone H2AX-like [Pyrus x bretschneideri] |
| EVM0022595.1 |  | PREDICTED: uncharacterized protein LOC103957072 isoform X1 [Pyrus x bretschneideri] |
| EVM0023050.1 |  | PREDICTED: bifunctional riboflavin kinase/FMN phosphatase-like [Pyrus x bretschneideri] |
| EVM0023112.1 |  | PREDICTED: LOW QUALITY PROTEIN: NADPH:adrenodoxin oxidoreductase, mitochondrial-like [Malus domestica] |
| EVM0023366.1 |  | PREDICTED: GDP-L-galactose phosphorylase 2-like isoform X1 [Pyrus x bretschneideri] |
| EVM0023398.1 | FRUC1 | PREDICTED: fructokinase-1 [Malus domestica] |
| EVM0023814.1 |  | PREDICTED: chaperonin CPN60-2, mitochondrial [Pyrus x bretschneideri] |
| EVM0024116.1 |  | PREDICTED: TMV resistance protein N-like [Pyrus x bretschneideri] |
| EVM0024423.1 |  | PREDICTED: putative E3 ubiquitin-protein ligase RF298 isoform X1 [Malus domestica] |
| EVM0024443.2 |  | PREDICTED: serine/arginine-rich splicing factor 4-like [Pyrus x bretschneideri] |
| EVM0024571.1 |  | PREDICTED: uncharacterized protein LOC103936662 [Pyrus x bretschneideri] |
| EVM0024647.1 |  | PREDICTED: TPR repeat-containing thioredoxin TDX-like [Pyrus x bretschneideri] |
| EVM0025299.1 |  | PREDICTED: phosphoglycerate kinase, cytosolic-like [Prunus mume] |
| EVM0025492.1 |  | PREDICTED: tRNA pseudouridine synthase-like 1 [Malus domestica] |
| EVM0025521.1 |  | PREDICTED: geraniol 8-hydroxylase-like [Pyrus x bretschneideri] |
| EVM0025705.1 | MET5 | PREDICTED: methyltransferase-like protein 5 [Pyrus x bretschneideri] |
| EVM0026221.1 |  | PREDICTED: myosin-9 [Malus domestica] |
| EVM0026920.1 |  | PREDICTED: midasin-like [Pyrus x bretschneideri] |
| EVM0027214.1 | SUVR2-like | PREDICTED: histone-lysine N-methyltransferase SUVR2-like [Pyrus x bretschneideri] |
| EVM0027216.1 |  | PREDICTED: glutamate--cysteine ligase, chloroplastic-like [Pyrus x bretschneideri] |
| EVM0027293.1 |  | PREDICTED: probable LRR receptor-like serine/threonine-protein kinase At3g47570 isoform X1 [Pyrus x bretschneideri] |
| EVM0027341.1 |  | PREDICTED: beta-amyrin 28-oxidase-like [Malus domestica] |
| EVM0027512.1 |  | PREDICTED: heat shock 70 kDa protein 17-like [Pyrus x bretschneideri] |
| EVM0027577.1 |  | PREDICTED: heparan-alpha-glucosaminide N-acetyltransferase-like [Pyrus x bretschneideri] |
| EVM0027594.1 |  | PREDICTED: uncharacterized protein LOC103947716 isoform X1 [Pyrus x bretschneideri] |
| EVM0027600.1 |  | PREDICTED: 3-ketoacyl-CoA synthase 21-like [Pyrus x bretschneideri] |
| EVM0027960.1 |  | PREDICTED: very-long-chain enoyl-CoA reductase-like [Pyrus x bretschneideri] |
| EVM0028030.1 |  | PREDICTED: vignain-like [Pyrus x bretschneideri] |
| EVM0028086.1 |  | PREDICTED: fanconi-associated nuclease 1 homolog isoform X1 [Pyrus x bretschneideri] |
| EVM0028185.1 |  | PREDICTED: (3S,6E)-nerolidol synthase 1-like [Pyrus x bretschneideri] |
| EVM0028273.1 |  | PREDICTED: DDT domain-containing protein DDB_G0282237 [Pyrus x bretschneideri] |
| EVM0028403.1 |  | PREDICTED: transcription factor WER-like [Pyrus x bretschneideri] |
| EVM0028453.1 |  | PREDICTED: receptor-like protein 12 [Malus domestica] |
| EVM0029070.1 |  | PREDICTED: tetraketide alpha-pyrone reductase 1-like isoform X1 [Pyrus x bretschneideri] |
| EVM0029199.1 | bHLH137-like | PREDICTED: transcription factor bHLH137-like isoform X1 [Pyrus x bretschneideri] |
| EVM0029370.1 |  | PREDICTED: polynucleotide 5&apos;-hydroxyl-kinase NOL9-like [Pyrus x bretschneideri] |
| EVM0029415.1 |  | PREDICTED: uncharacterized protein LOC103408204 [Malus domestica] |
| EVM0029728.1 |  | PREDICTED: uncharacterized protein LOC103966906 isoform X3 [Pyrus x bretschneideri] |
| EVM0029818.1 |  | PREDICTED: lon protease homolog 1, mitochondrial-like [Malus domestica] |
| EVM0029844.1 |  | PREDICTED: uncharacterized protein LOC103424534 [Malus domestica] |
| EVM0029892.1 |  | PREDICTED: uncharacterized protein LOC103451435 [Malus domestica] |
| EVM0030130.1 |  | PREDICTED: uncharacterized protein At3g06530-like [Pyrus x bretschneideri] |
| EVM0030450.1 |  | PREDICTED: uncharacterized protein LOC103957697 [Pyrus x bretschneideri] |
| EVM0030516.1 | HDT2 | PREDICTED: histone deacetylase HDT2-like [Pyrus x bretschneideri] |
| EVM0030835.1 |  | PREDICTED: uncharacterized protein LOC103943452 [Pyrus x bretschneideri] |
| EVM0031035.1 |  | PREDICTED: protein prenyltransferase alpha subunit repeat-containing protein 1 [Malus domestica] |
| EVM0031103.1 |  | PREDICTED: uncharacterized protein LOC103327233 [Prunus mume] |
| EVM0031163.1 |  | PREDICTED: uncharacterized protein LOC103416498 [Malus domestica] |
| EVM0031245.1 |  | PREDICTED: uncharacterized protein LOC103949825 [Pyrus x bretschneideri] |
| EVM0031268.1 |  | PREDICTED: calcineurin B-like protein 7 [Pyrus x bretschneideri] |
| EVM0031604.1 |  | PREDICTED: probable nucleoside diphosphate kinase 5 isoform X1 [Pyrus x bretschneideri] |
| EVM0031832.1 |  | PREDICTED: probable ADP-ribosylation factor GTPase-activating protein AGD15 isoform X1 [Pyrus x bretschneideri] |
| EVM0031998.1 |  | PREDICTED: serine/threonine-protein kinase mph1 isoform X2 [Pyrus x bretschneideri] |
| EVM0032120.1 |  | PREDICTED: uncharacterized protein LOC103942441 isoform X1 [Pyrus x bretschneideri] |
| EVM0032192.1 |  | PREDICTED: uncharacterized protein LOC103944764 [Pyrus x bretschneideri] |
| EVM0032346.1 |  | PREDICTED: phospholipase A(1) LCAT3-like [Malus domestica] |
| EVM0032482.1 |  | PREDICTED: frataxin, mitochondrial-like [Pyrus x bretschneideri] |
| EVM0032503.1 |  | PREDICTED: mediator of RNA polymerase II transcription subunit 1-like [Pyrus x bretschneideri] |
| EVM0032638.1 |  | PREDICTED: novel plant SNARE 11-like isoform X1 [Pyrus x bretschneideri] |
| EVM0032865.1 |  | PREDICTED: DNA repair protein RAD50 [Pyrus x bretschneideri] |
| EVM0033077.1 |  | PREDICTED: DNA-directed primase/polymerase protein-like [Pyrus x bretschneideri] |
| EVM0033078.1 |  | PREDICTED: uncharacterized protein LOC103947266 isoform X2 [Pyrus x bretschneideri] |
| EVM0033100.1 |  | PREDICTED: receptor-like protein 2 isoform X1 [Malus domestica] |
| EVM0033484.1 |  | PREDICTED: uncharacterized protein LOC103931485 [Pyrus x bretschneideri] |
| EVM0033777.1 |  | PREDICTED: ATPase family AAA domain-containing protein 3-like [Pyrus x bretschneideri] |
| EVM0034113.1 |  | PREDICTED: probable cytokinin riboside 5&apos;-monophosphate phosphoribohydrolase LOGL1 [Pyrus x bretschneideri] |
| EVM0034791.1 |  | PREDICTED: U3 small nucleolar ribonucleoprotein protein IMP4-like [Malus domestica] |
| EVM0035230.1 |  | PREDICTED: uncharacterized protein LOC103949011 [Pyrus x bretschneideri] |
| EVM0035383.1 |  | PREDICTED: uncharacterized protein LOC103933850 [Pyrus x bretschneideri] |
| EVM0035411.1 |  | PREDICTED: pentatricopeptide repeat-containing protein At3g48250, chloroplastic-like [Pyrus x bretschneideri] |
| EVM0035542.1 |  | PREDICTED: uncharacterized protein LOC103438970 [Malus domestica] |
| EVM0035597.1 |  | PREDICTED: B3 domain-containing protein Os01g0234100-like isoform X2 [Pyrus x bretschneideri] |
| EVM0035615.1 |  | PREDICTED: probable LRR receptor-like serine/threonine-protein kinase At5g45780 [Pyrus x bretschneideri] |
| EVM0035619.1 | GBF1 | PREDICTED: G-box-binding factor 1-like [Pyrus x bretschneideri] |
| EVM0035919.1 |  | PREDICTED: peptide chain release factor 1-like, mitochondrial isoform X1 [Pyrus x bretschneideri] |
| EVM0036095.1 |  | PREDICTED: uncharacterized protein LOC103935321 [Pyrus x bretschneideri] |
| EVM0036419.1 |  | PREDICTED: sodium-dependent phosphate transport protein 1, chloroplastic-like [Pyrus x bretschneideri] |
| EVM0036993.1 |  | PREDICTED: DNA-directed RNA polymerase V subunit 5C [Pyrus x bretschneideri] |
| EVM0037027.1 |  | PREDICTED: tropomyosin alpha-1 chain-like [Malus domestica] |
| EVM0037167.1 |  | PREDICTED: uncharacterized protein LOC103941626 [Pyrus x bretschneideri] |
| EVM0037425.1 |  | PREDICTED: uncharacterized protein LOC103963519 [Pyrus x bretschneideri] |
| EVM0037807.1 |  | PREDICTED: protein WHI4-like [Malus domestica] |
| EVM0037847.1 |  | PREDICTED: histone H2AX [Malus domestica] |
| EVM0037912.1 |  | PREDICTED: uncharacterized protein LOC103943905 [Pyrus x bretschneideri] |
| EVM0038614.1 |  | PREDICTED: protoheme IX farnesyltransferase, mitochondrial-like [Pyrus x bretschneideri] |
| EVM0039498.1 |  | PREDICTED: adenosine kinase-like [Pyrus x bretschneideri] |
| EVM0039954.1 |  | PREDICTED: E3 ubiquitin-protein ligase MIB2-like [Malus domestica] |
| EVM0040123.1 |  | PREDICTED: 60S ribosomal protein L3-2-like [Pyrus x bretschneideri] |
| EVM0040348.1 |  | PREDICTED: uncharacterized protein LOC103929339 [Pyrus x bretschneideri] |
| EVM0040400.1 |  | PREDICTED: ABC transporter C family member 8 [Pyrus x bretschneideri] |
| EVM0040475.1 |  | PREDICTED: BAHD acyltransferase At5g47980-like [Pyrus x bretschneideri] |
| EVM0040849.1 | SUS2 | PREDICTED: sucrose synthase 2-like [Pyrus x bretschneideri] |
| EVM0040870.1 |  | hypothetical protein PRUPE_ppa005529mg [Prunus persica] |
| EVM0040892.1 |  | PREDICTED: uncharacterized protein LOC103951924 [Pyrus x bretschneideri] |
| EVM0040929.1 |  | PREDICTED: ABC transporter G family member 17-like [Malus domestica] |
| EVM0040978.1 |  | PREDICTED: LOW QUALITY PROTEIN: neuroguidin-like [Malus domestica] |
| EVM0041019.1 |  | #N/A |
| EVM0041401.1 |  | PREDICTED: protein NRT1/ PTR FAMILY 6.3-like [Pyrus x bretschneideri] |
| EVM0041437.1 | ARF18 | PREDICTED: auxin response factor 18 [Malus domestica] |
| EVM0042087.1 |  | PREDICTED: probable inactive receptor kinase At5g58300 [Pyrus x bretschneideri] |
| EVM0042193.1 | TCP4 | PREDICTED: transcription factor TCP4-like [Pyrus x bretschneideri] |
| EVM0042241.1 |  | PREDICTED: LOW QUALITY PROTEIN: aconitate hydratase, cytoplasmic [Pyrus x bretschneideri] |
| EVM0042280.1 |  | PREDICTED: nucleolar complex protein 2 homolog [Pyrus x bretschneideri] |
| EVM0042313.1 |  | PREDICTED: uncharacterized protein LOC103439620 [Malus domestica] |
| EVM0042378.1 | bHLH90 | PREDICTED: transcription factor bHLH90 [Malus domestica] |
| EVM0042405.1 | bHLH91-like | PREDICTED: transcription factor bHLH91-like [Pyrus x bretschneideri] |
| EVM0042445.1 |  | PREDICTED: serine--tRNA ligase, mitochondrial-like [Pyrus x bretschneideri] |

**Table S6 Summary of *DAM* genes in this study and previous study**

| **Gene names in this study** | **Gene accession in ‘Cuiguan’ genome** | **NCBI accession NO.** | **Gene names in previous studies** | | |
| --- | --- | --- | --- | --- | --- |
| DAM1 | EVM0015109 | XP_009376231.1  XP_009376233.1 | DAM1 |  | MADS13-2 |
| DAM2 | EVM0009473 | XP_009376230.1 |  |  | MADS13-3 |
| DAM3 | EVM0041302 | XP_009376226.1  XP_009376227.1 |  |  |  |
| DAM4-1  DAM4-2 | EVM0039919  EVM0004250 | XP_018500348.1 | DAM2  DAM3 | DAM1 | MADS13-1 |
| **Reference** |  |  | Niu et al. 2016; Yang et al. 2020 | Tuan et al. 2017 | Ubi et al. 2010; Saito et al. 2013 |

**Table S7 Accession numbers of genes used in phylogenetic analysis of DAM proteins**

| Gene | Accession number | Source |
| --- | --- | --- |
| AdSVP-1 | AFA37963.1 | NCBI |
| AdSVP-2 | AFA37964.1 | NCBI |
| AdSVP-3 | AFA37965.1 | NCBI |
| AdSVP-4 | AFA37966.1 | NCBI |
| AtAGL24 | NP_194185.1 | NCBI |
| AtSVP | NP_001324584.1 | NCBI |
| PtSVP-1 | RQO90600.1 | NCBI |
| PtSVP-2 | XP_024461638.1 | NCBI |
| PtSVP-3 | XP_002310310.1 | NCBI |
| VvSVP-1 | QBC35958.1 | NCBI |
| VvSVP-2 | XP_002285687.1 | NCBI |
| VvSVP-3 | XP_019073897.1 | NCBI |
| PpeDAM1 | ABJ96361.2 | NCBI |
| PpeDAM2 | ABJ96370.1 | NCBI |
| PpeDAM3 | ABJ96371.1 | NCBI |
| PpeDAM4 | ABJ96358.1 | NCBI |
| PpeDAM5 | ABJ96366.1 | NCBI |
| PpeDAM6 | ABJ96360.1 | NCBI |
| MdSVP-1 | MDP0000209705 | GDR |
| MdSVP-2 | MDP0000132226 | GDR |
| MdSVP-3 | MDP0000259294 | GDR |
| MdSVP-4 | MDP0000259296 | GDR |
| MdSVP-5 | MDP0000277582 | GDR |
| MdSVP-6 | MDP0000527190 | GDR |
| MdSVP-7 | MDP0000233948 | GDR |
| MdSVP-8 | MDP0000255146 | GDR |
| MdSVP-9 | MDP0000378412 | GDR |
| MdSVP-10 | MDP0000322567 | GDR |
| MdSVP-11 | MDP0000223851 | GDR |
| MdSVP-12 | MDP0000143531 | GDR |
| MdSVP-13 | MDP0000311107 | GDR |
| FvSVP-1 | gene03714-v1.0-hybrid | GDR |
| FvSVP-2 | gene03748-v1.0-hybrid | GDR |
| FvSVP-3 | gene20808-v1.0-hybrid | GDR |
| FvSVP-4 | gene12120-v1.0-hybrid | GDR |
| FvSVP-5 | gene12119-v1.0-hybrid | GDR |
| PpeSVP-1 | Prupe.8G069300.2 | GDR |
| PpeSVP-2 | Prupe.6G199000.5 | GDR |
| PpyDAM1 | EVM0015109.1 | This article |
| PpyDAM2 | EVM0009473.1 | This article |
| PpyDAM3 | EVM0041302.1 | This article |
| PpyDAM4-1 | EVM0039919.1 | This article |
| PpyDAM4-2 | EVM0004250.1 | This article |

**Table S8 Primers used for qRT-PCR**

| Gene name | Forward primer (5’ ~ 3’) | Reverse primer (5’ ~ 3’) |
| --- | --- | --- |
| Q-Cyclin-J18 | CGCTTGTTTCGCTGTGGATT | GTTCCCCAACTTTTGCCACC |
| Q-DAM1 | TCGAAGATGACTGCTCCGAC | ACAGTCACAGTACTTCCACAGA |
| Q-DAM2 | GAAGAAGGCGTGACATCTGAATC | CCATCCGTCTCCACTTCTTAACCAA |
| Q-DAM3 | GACGGCGATCAAGGAGTT | CAAGTTAAGGCGGCTCACC |
| Q-DAM4 | AACAACCAGCTAAGCCAGAAG | TCAAGGGTTGGAGAAGGTGG |
| Q-EXPA1 | CGAAACTGGGGGCAAAACTG | AAATTAGGAGGTGGCGTGGG |
| Q-FY | TGGGCTCGCTCCTCCTTAT | AGGCAACTGACGGCAACATA |
| Q-GA20OX2 | GGCCCTGGCTTTTCACTCATC | GGAGGTTGTGTCATGGTTTGC |
| Q-GH3 | CGGACTTCTTGGTCTCCCTC | CGTAAGTGGCGACAGTGCT |
| Q-LAC12 | ACACCAGTGAAGAGGCTGTG | TCCCTCCTGGCCTAATTGGA |
| Q-MYB108 | TGGAACATGGACGGGATGTG | GCAGTTTGAATACATCCCCTAACC |
| Q-NAC88 | GGTGGACAACTCGGGATTCA | CCCTCTCTACCGCCCTTTTG |
| Q-SDH | TGGAAGTCATCGTCTCGGATG | GCATACGTACGCACACAATTATTA |
| Q-TCP4 | GGGATTCTCACCACCACCAAG | CACGATGTCGGTGCTGACTA |
| Q-TIFY10A | CCCTCGACCCGTTGTTTGT | GTTTGGTACGGTGCTAGGGT |

**Table S9 Primers used for VIGS experiment**

| Primer name | Primer sequence |
| --- | --- |
| DAM-pTRV2-F | ATTCTGTGAGTAAGGTTACCGATTGGTGGAAGCAAGCCTTGGCCG |
| DAM-pTRV2-R | GCCCGGGCCTCGAGACGCGTGGGAGAAGGTGGTGACATTTGTGGC |
